# Oral typhoid vaccine Ty21a elicits antigen-specific resident memory CD4^+^ T cells in the human terminal ileum lamina propria and epithelial compartments

**DOI:** 10.1186/s12967-020-02263-6

**Published:** 2020-02-25

**Authors:** Jayaum S. Booth, Eric Goldberg, Robin S. Barnes, Bruce D. Greenwald, Marcelo B. Sztein

**Affiliations:** 1 Center for Vaccine Development and Global Health, University of Maryland School of Medicine, Baltimore, MD 21201 USA; 2 Department of Pediatrics, University of Maryland School of Medicine, Baltimore, MD USA; 3 Department of Medicine, University of Maryland School of Medicine, Baltimore, MD USA; 4 Division of Gastroenterology and Hepatology, University of Maryland School of Medicine, Baltimore, MD USA

**Keywords:** Tissue resident CD4^+^ T cells, Terminal ileum LPMC, IEL, Ty21a, Oral vaccine

## Abstract

**Background:**

*Salmonella* enterica serovar Typhi (*S*. Typhi) is a highly invasive bacterium that infects the human intestinal mucosa and causes ~ 11.9–20.6 million infections and ~ 130,000–223,000 deaths annually worldwide. Oral typhoid vaccine Ty21a confers a moderate level of long-lived protection (5–7 years) in the field. New and improved vaccines against enteric pathogens are needed but their development is hindered by a lack of the immunological correlates of protection especially at the site of infection. Tissue resident memory T (T_RM_) cells provide immediate adaptive effector immune responsiveness at the infection site. However, the mechanism(s) by which *S*. Typhi induces T_RM_ in the intestinal mucosa are unknown. Here, we focus on the induction of *S.* Typhi-specific CD4+T_RM_ subsets by Ty21a in the human terminal ileum lamina propria and epithelial compartments.

**Methods:**

Terminal ileum biopsies were obtained from consenting volunteers undergoing routine colonoscopy who were either immunized orally with 4 doses of Ty21a or not. Isolated lamina propria mononuclear cells (LPMC) and intraepithelial lymphocytes (IEL) CD4+T_RM_ immune responses were determined using either *S*. Typhi-infected or non-infected autologous EBV-B cell lines as stimulator cells. T-CMI was assessed by the production of 4 cytokines [interferon (IFN)γ, interleukin (IL)-2, IL-17A and tumor necrosis factor (TNF)α] in 36 volunteers (18 vaccinees and 18 controls volunteers).

**Results:**

Although the frequencies of LPMC CD103+ CD4+T_RM_ were significant decreased, both CD103+ and CD103− CD4+T_RM_ subsets spontaneously produced significantly higher levels of cytokines (IFNγ and IL-17A) following Ty21a-immunization. Importantly, we observed significant increases in *S*. Typhi-specific LPMC CD103+ CD4+T_RM_ (IFNγ and IL-17A) and CD103− CD4+T_RM_ (IL-2 and IL-17A) responses following Ty21a-immunization. Further, differences in *S*. Typhi-specific responses between these two CD4+T_RM_ subsets were observed following multifunctional analysis. In addition, we determined the effect of Ty21a-immunization on IEL and observed significant changes in the frequencies of IEL CD103+ (decrease) and CD103− CD4+T_RM_ (increase) following immunization. Finally, we observed that IEL CD103− CD4+T_RM_, but not CD103+ CD4+T_RM_, produced increased cytokines (IFNγ, TNFα and IL-17A) to *S*. Typhi-specific stimulation following Ty21a-immunization.

**Conclusions:**

Oral Ty21a-immunization elicits distinct compartment specific immune responses in CD4+T_RM_ (CD103+ and CD103−) subsets. This study provides novel insights in the generation of local vaccine-specific responses.

*Trial registration* This study was approved by the Institutional Review Board and registered on ClinicalTrials.gov (identifier *NCT03970304*, Registered 29 May 2019—Retrospectively registered, http://www.ClinicalTrials.gov/NCT03970304)

## Background

CD4+ T cells are crucial for the generation of vaccine-mediated immune responses providing effective immunity against pathogens. Recent studies have demonstrated that both circulating T memory (T_M_) cells and tissue resident memory T cells (T_RM_) abundant in peripheral tissues are central to elicit protective immunity [1, 2]. T_RM_ represent a non-migratory population of T_M_ that is phenotypically different from circulating T_M_ subsets [e.g., T central memory (T_CM_) and T effector memory (T_EM_)] and mediate rapid effector immune responses following antigen recall [2]. Human T_RM_ are mainly characterized phenotypically by high expression of CD69, a key marker for distinguishing between circulating and resident T_M_ [3]. Integrin αEβ7 (CD103), the ligand to E-cadherin, is also used to characterize T_RM_ but CD103 expression is mostly confined to CD8+ T_RM_ and a minor subset of CD4+T_RM_ [3–6]. Recent studies have characterized CD4+ T_RM_ subsets in multiple organs including lungs, liver, skin, intestines, vagina and lymphoid sites where they provide protective responses and direct the recruitment of immune cells [7–10]. In the human intestine, the majority of CD4+T_RM_ are CD69+CD103− and a minority are CD69+CD103+ [3] but little information is available concerning their role in oral immunization or enteric infections. It is unclear whether live oral attenuated typhoid vaccine, Ty21a, could elicit and generate antigen-specific CD4+T_RM_ responses in the human terminal ileum (TI) (site of infection for *S*. Typhi).

*Salmonella enterica* serovar Typhi (*S*. Typhi), the etiological agent of typhoid fever, is a human restricted pathogen, that infects around 21 million people globally resulting in approximately to 223,000 deaths yearly [11–13]. After actively infecting primarily the TI [14–16], *S*. Typhi disseminate to the systemic organs (e.g., liver, spleen and others) leading to systemic illness [17]. To our knowledge, no information is available on the induction of CD4+T_RM_ responses to *S.* Typhi in the TI mucosa following wild-type *S*. Typhi infection or immunization with Ty21a. Since the gastrointestinal track is a major reservoir of CD4+T_RM_, it is important to understand their role in protective immunity against *S*. Typhi and other enteric pathogens at their preferred site of natural infection. This knowledge will provide novel insights for the development of tailored mucosal vaccines to enteric pathogens. Currently, there are two licensed typhoid vaccines namely, Ty21a and the parenteral Vi polysaccharide vaccine [18, 19] and both have their limitations. Following Ty21 immunization, a modest level of long-lived protection (60–80%, 5–7 years) is obtained [20–22] while the Vi vaccine is moderately immunogenic but well tolerated [17, 23]. Therefore, effective vaccines that offer durable, long-lasting protection are urgently needed for children and adults.

Induction of humoral and CMI responses have been thoroughly examined in peripheral blood mononuclear cells (PBMC) obtained from healthy individuals vaccinated with Ty21a [24–30]. These studies demonstrated that both CD4+ and CD8+ T cell responses (e.g., IFNγ, cytotoxic T cells (CTL), proliferation) are induced following Ty21a-vaccination [22, 24, 25, 27, 31, 32]. Moreover, *S*. Typhi responsive CD4+ T cells are mediated mostly by T_EM_ and RA+T_EM_ (T_EMRA_) and, to a lesser extent, by T_CM_ [22, 32, 33]. Furthermore, CD4+T_EM_ and T_EMRA_ subsets responses displayed increases in *S.* Typhi-specific multifunctional (MF) cells post-vaccination mainly producing IFN-γ and/or TNF-α, while IL-2, MIP-1β, IL-17A and CD107a expression (a marker associated with cytotoxicity) were produced in a small proportion of MF cells [33]. Recently, we reported that oral Ty21a-immunization elicits significant TI lamina propria mononuclear cells (LPMC) *S*. Typhi-specific CD8+ T_M_ [34], CD8+T_RM_ [16], and CD4+T_M_ [35] responses with distinct effector functions. However, it is unknown whether LPMC CD4+T_RM_ are elicited following oral Ty21a immunization.

Likewise, we have characterized intraepithelial lymphocytes (IEL) CD8+T_RM_*S*. Typhi specific responses in the epithelium compartment following Ty21a-immunization [16]. Interestingly, no information is available on the role of the IEL CD4+T_RM_ following oral Ty21a-vaccination. Functional studies with human intestinal IEL are challenging due to their low yield during the isolation process. Nonetheless, IEL are among the first line of defense and it is therefore important to understand their role in oral Ty21a-vaccination and *S*. Typhi infection.

In this study, we have characterized TI LPMC and IEL CD4+T_RM_ subsets following Ty21a immunization. We then determined and compared CD4+T_RM_*S*. Typhi-specific responses between the two groups. Finally, we assessed single and multifunctional *S*. Typhi specific responses in CD4+ T_RM_ subsets by single cell analysis. These comparisons provide unique insights into the generation of *S*. Typhi specific responses in the human TI mucosa.

## Methods

### Volunteers, immunization and sample collection

Individuals (aged 50–74 years) undergoing routine colonoscopy were enrolled from the Baltimore–Washington metropolitan area and University of Maryland, Baltimore campus. Volunteers who have no previous history of typhoid fever and were not vaccinated with the attenuated Ty21a typhoid vaccine were assigned to each of two groups. Four recommended doses of Ty21a (Vivotif enteric-coated capsules; Crucell, Bern, Switzerland) were administrated to the first group (n = 18) but not to the control group (n = 18) (Additional file 1: Fig. S1). Blood samples were collected at least 21 days before colonoscopy (pre-immunization) and on colonoscopy day (day 0) together with TI biopsies using large capacity forceps (Additional file 1: Fig. S1). PBMC were isolated immediately after blood draws by density gradient centrifugation and cryopreserved in liquid nitrogen following standard techniques [32].

### Ethics statement

Written informed consent was obtained from subjects and all procedures were approved by the University of Maryland, Baltimore Institutional Review Board (IRB) and registered on ClinicalTrials.gov (identifier NCT03970304). The study was conducted in accordance with the principles of the International Conference of Harmonization Good Clinical Practice guidelines.

### Isolation of lamina propria mononuclear cells (LPMC) and intraepithelial lymphocytes (IEL) from terminal ileum biopsies

TI-LPMC and IEL were freshly isolated as previously described [16, 36–38]. Briefly, after collection of biopsies from volunteers undergoing routine colonoscopy, tissues were treated with HBSS (without CaCl_2_, MgCl_2_, MgSO_4_; Gibco, Carlsbad, CA) and EDTA (10 mM; Ambion, Grand Island, NY) and were vigorously shaken for 45 min to isolate IEL. Next, the biopsies were digested enzymatically with collagenase D (100 μg/mL; Roche, Indianapolis, IN) and DNase I (10 μg/mL; Affymetrix, Cleveland, OH) for 45 min followed by homogenization using the Bullet Blender homogenizer (Next Advance Inc, Averill, NY) to extract LPMC. LPMC and IEL were either stained immediately for immunophenotyping by flow cytometry or stimulated overnight.

### Target cell preparation and *S*. Typhi infection

Autologous Epstein–Barr virus (EBV)-transformed lymphoblastoid cell line (EBV-B cells) were generated from each participant’s pre-immunized PBMC (Additional file 1: Fig. S1) as previously described [25, 32]. Target cells were then infected with wt-*S.* Typhi strain ISP1820 at a MOI of 7:1 as previously described [35]. Infected target cells were then gamma-irradiated (6000 rad) before used for ex vivo LPMC and IEL stimulation. To confirm *S*. Typhi infection, target cells were stained with anti-*Salmonella* common structural Ag (CSA-1, Kierkegaard and Perry, Gaithersburg, MD) and analyzed by flow cytometry as previously described [25, 32].

### Stimulation of terminal ileum LPMC and IEL

Freshly isolated TI-LPMC and IEL were used as effector cells as previously described [16, 34, 37]. Briefly, LPMC and IEL were co-cultured, respectively, with either un-infected or *S*. Typhi-infected EBV-B (MOI of 7:1). LPMC and IEL cultured with media only or in the presence of α-CD3/CD28 (Life technologies, Grand Island, NY) were used as negative and positive controls, respectively. After 2 h, 0.5 μL of Golgi Stop (Monensin, BD) and 0.5 μL Golgi Plug (Brefeldin A, BD) were added and cultures continued overnight at 37 °C in 5% CO_2_.

### Surface and intracellular staining

Following overnight stimulation, TI LPMC and IEL were stained for flow cytometry analysis as previously described [16]. Briefly, LPMC and IEL were stained for live/dead discrimination (YEVID) (Invitrogen, Carlsbad, CA) and then the Fc receptors were blocked using human immunoglobulin (3 μg/mL; Sigma). This was followed by surface staining. Briefly, cells were stained at 4 °C for 30 min with fluorescently labeled monoclonal antibodies directed to CD13-Pacific Orange (conjugated in-house), CD19-BV570 (HIB19, Biolegend, San Diego, CA), CD3-BV650 (OKT3, Biolegend), CD4-PE-Cy5 (RPA-T4, BD), CD8-PerCP-Cy5.5 (SK1, BD), CD45RA-biotin (HI100, BD), CD62L-APC-A780 (DREG-56, eBioscience, San Diego, CA), and CD103-FITC (Ber-ACT8, BD). After a wash, cells were stained with streptavidin (SAV)-Qdot800 (Invitrogen) at 4 °C for 30 min. Cells were then fixed and permeabilized using IC fixation and permeabilization buffers (8222/8333, eBioscience). This was followed by staining with monoclonal antibodies directed to interleukin (IL)-17A-BV421 (BL168, Biolegend), interferon (IFN)-γ-PE-Cy7 (B27, BD), tumor necrosis factor (TNF)-α-Alexa 700 (MAb11, BD), and CD69-ECD (TP1.55.3, Beckman Coulter, Danvers, MA), and IL2-BV605 (MQ1-17H12, Biolegend). After staining, cells were stored in 1% paraformaldehyde at 4 °C until data collection. Data were collected using a customized LSRII flow cytometer (BD) and then analyzed using the WinList version 7 (Verity Software House, Topsham, ME) software package. *S.* Typhi-specific responses were expressed as net percentage of positive cells (background after stimulation with uninfected cells were subtracted from values obtained with *S.* Typhi-infected targets). The FCOM function of WinList was used to determine *S.* Typhi-specific MF responses in TI LPMC and IEL. Flow cytometry experiments were performed at the Flow Cytometry and Mass Cytometry Core Facility of the University of Maryland School of Medicine Center for Innovative Biomedical Resources (CIBR), Baltimore, Maryland.

### Statistical analysis

Data were analyzed using the statistical software GraphPad Prism™ version 5.03 (Graphpad, San Diego, CA, USA). Statistical differences in median values between two groups were determined using Mann–Whitney tests. Wilcoxon matched pair tests were used to assess statistical differences between LPMC and IEL paired responses. Correlations (LPMC versus IEL *S.* Typhi-specific responses) were evaluated using Spearman correlation tests.

### Data availability

The datasets supporting the findings of this study are available within the article and its additional information files.

## Results

### Oral Ty21a-immunization influences the frequencies of terminal ileum CD4+T_RM_ subsets

**Fig. 1:**
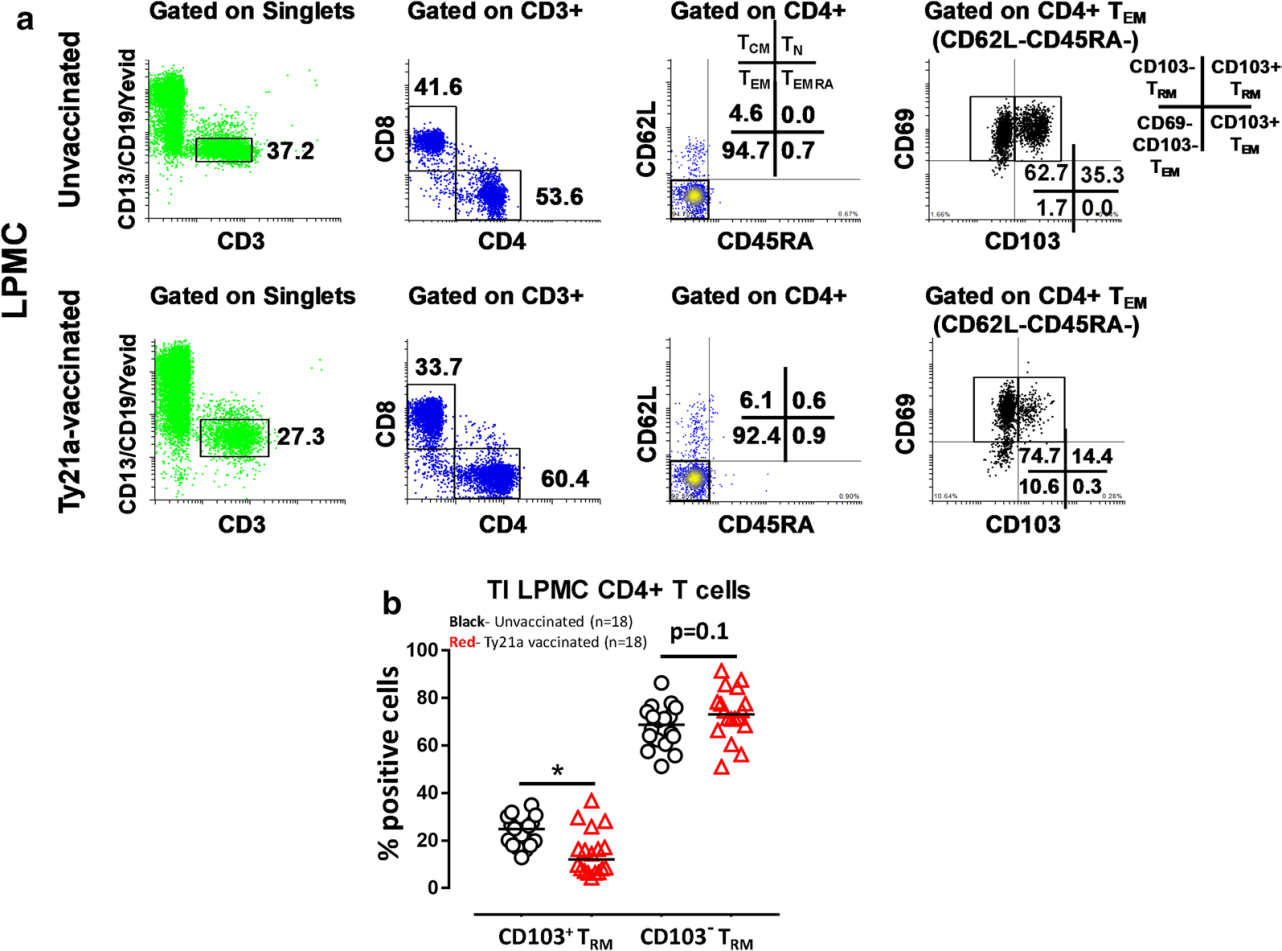
Gating Strategy and cell subset frequencies of terminal ileum tissue-resident memory CD4+ T (T_RM_) cells. Terminal ileum LPMC CD4+ tissue resident T memory cell (T_RM_) subsets in representative volunteers were detected in **a** Unvaccinated and Ty21a-vaccinated participants by expression of CD69 and CD103 markers following the gating strategy shown in the figure. **b** Frequencies of CD4+ CD103+ T_RM_ and CD4+ CD103− T_RM_ subsets were measured and compared between TI LPMC obtained from Ty21a-vaccinated (n = 18; red symbols) and unvaccinated volunteers (n = 18; black symbols) with significant differences (*p < 0.05) and a trend is indicated by its p-value. Horizontal bars represent median values

Recent evidence suggest that CD4+T_RM_ may influence local immune responses at the site of infection [7, 9]. However, the effect of oral Ty21a-immunization on TI CD4+T_RM_ is unknown. To explore this phenomenon, we characterized CD4+T_RM_ subsets (CD103+ and CD103−) from freshly isolated TI-LPMC obtained from biopsies of Ty21a-vaccinated and unvaccinated volunteers (Fig. 1a). As expected, using CD69 and CD103 markers, TI-LPMC CD4+T_RM_ were comprised of two populations, namely CD69+CD103− (~ 70%) and CD69+CD103+ (~ 20%) (Fig. 1a). As observed in the representative volunteers (Fig. 1a), cumulative data demonstrated that following Ty21a-immunization, CD103+CD4+T_RM_ frequencies were significantly decreased (p < 0.05) whereas CD103− CD4+T_RM_ frequencies showed a trend to exhibit increased frequencies (p = 0.1; Fig. 1b), indicating that Ty21a-immunization affects the frequencies of TI-LPMC CD4+T_RM_. As expected, virtually no CD4+T_RM_ were observed in PBMC isolated from the same volunteers (Additional file 2: Fig. S2).

### Activation of terminal ileum LPMC CD4+T_RM_

CD4+T cells is the principal population in the TI lamina propria (LP) comprising most of the local CD4+T_RM_ (Fig. 1a). The observation that oral Ty21a-immunization influences CD4+T_RM_ frequencies (Fig. 1b) suggests that TI-LPMC CD4+T_RM_ subsets might respond differently in magnitude and characteristics following stimulation. To test this hypothesis, we determined the cytokine production by LPMC CD103+ and CD103− CD4+T_RM_ obtained from Ty21a-vaccinated (n = 18) and unvaccinated (n = 18) volunteers following co-culture with autologous *S*. Typhi-infected or uninfected EBV-B (targets) cells. Responses of a representative subject (Ty21a vaccinated) for CD103+ (Additional file 3: Fig. S3A) and CD103− (Additional file 3: Fig. S3B) CD4+T_RM_ are shown. Following stimulation with *S*. Typhi-infected targets, we observed substantial net increases (% of *S.* Typhi-infected EBV-B responses—% of uninfected EBV-B responses) in CD103+ CD4+T_RM_ cytokines (i.e., INFγ, 1.2%; IL-17A, 0.9%; IL-2, 0.3%; TNFα, 0.4%) producing cells in this representative Ty21a-vaccinee (Additional file 3: Fig. S3A). We also assessed LPMC CD103− CD4+T_RM_ responses and observed substantial net increases in CD103− CD4+T_RM_ producing cytokines (i.e., INFγ, 0.3%; IL-17A, 0.6%; IL-2, 0.7%; TNFα, 0.2%) (Additional file 3: Fig. S3B) frequencies. Therefore, we observed that both CD4+T_RM_ subsets are responsive to *S*. Typhi-stimulation following oral Ty21a immunization.

**Fig. 2:**
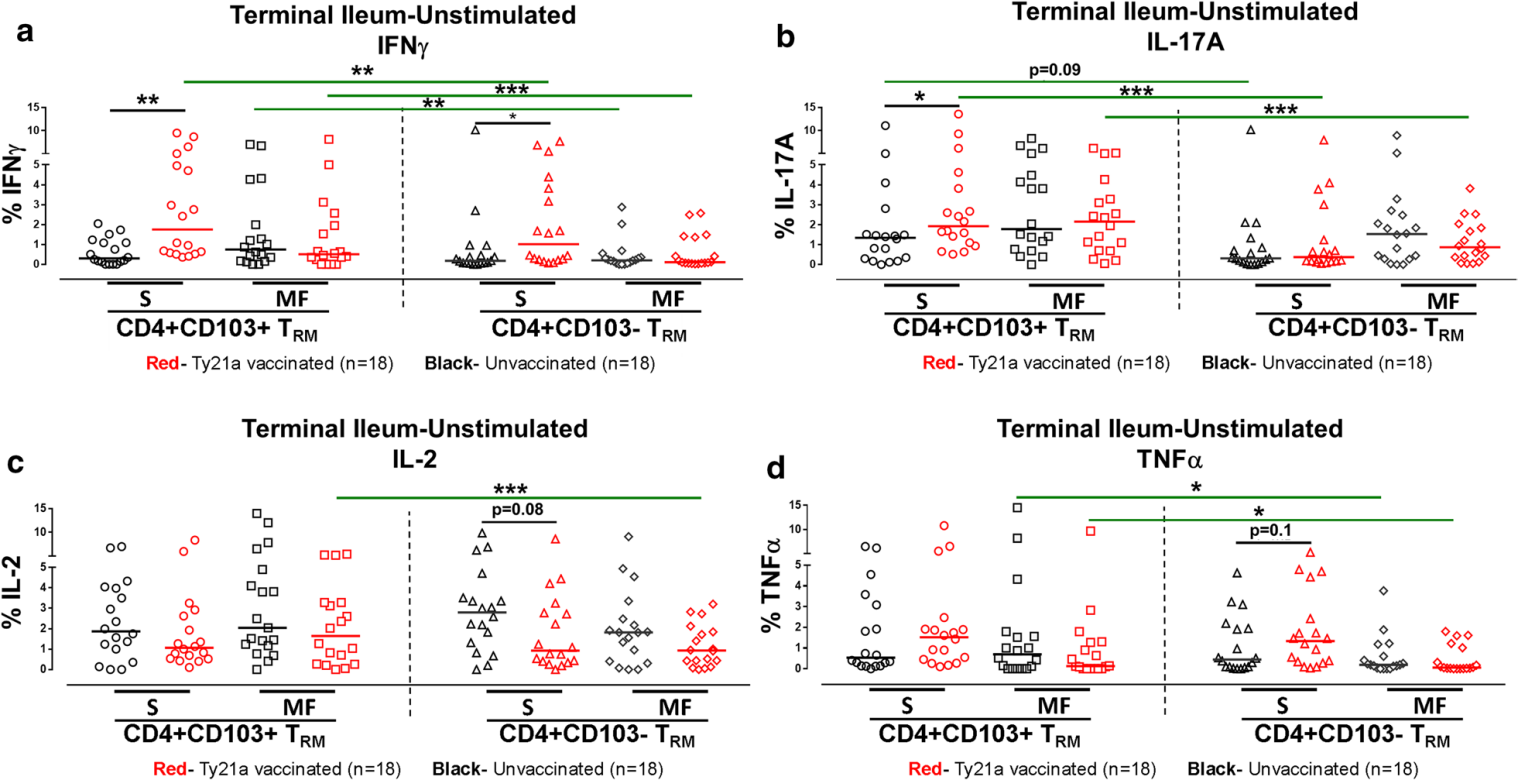
Effect of oral Ty21a-immunization on ex-vivo terminal ileum LPMC CD4+CD103+ and CD4+CD103− T_RM_. Ex-vivo unstimulated CD4+CD103+ and CD4+CD103− T_RM_ were cultured overnight and their levels of spontaneous cytokine (IFNγ, IL-17A, IL-2 and TNFα) production determined by flow cytometry. Using the FCOM function of Winlist, CD4+CD103+ and CD8+CD103− T_RM_ responses were stratified into single-positive cells (S) and multifunctional cells (MF). Comparison of TI LPMC CD4+ CD103+ and CD4+ CD103− T_RM_ responses in **a** INFγ+; **b** IL-17A+; **c** IL-2+, and **d** TNFα+ S and MF in Ty21a-vaccinated (n = 18; red symbols) and unvaccinated volunteers (n = 18; black symbols) with significant differences shown (*p < 0.05; **p < 0.005; ***p < 0.0005). Black lines: differences between Ty21a vaccinated and unvaccinated volunteers. Green lines: differences between CD4+CD103+ and CD4+CD103− T_RM_ cell responses. Trends are represented by their p-values. Horizontal bars (black and red) represent median values

Interestingly, we noted that both CD4+T_RM_ subsets exhibited differences in the background (unstimulated) and α-CD3/CD28 stimulation responses between Ty21a-vaccinated and unvaccinated volunteers. Therefore, we hypothesized that Ty21a-immunization might influence the induction of spontaneous cytokines (background responses) and the capacity of CD4+T_RM_ to respond to stimulation. To address this hypothesis, we evaluated CD4+T_RM_ (CD103+ and CD103−) cytokines producing cells when LPMC cells were cultured overnight either alone (unstimulated) or following stimulation with α-CD3/CD28 beads. Cumulative data from unstimulated LPMC show that there were significant (p < 0.05) increase in IFNγ-S (but not IFNγ-MF) producing cells in both CD4+T_RM_ subsets (Fig. 2a). Interestingly, we observed that the baseline level of IFNγ producing cells by CD103+ CD4+T_RM_ cells were significantly higher than those from CD103− CD4+T_RM_ as shown by green lines (Fig. 2a). Next, we determined spontaneous IL-17A responses and observed that CD103+ CD4+T_RM_ exhibited significantly higher levels of IL-17A-S following Ty21a-immunization (Fig. 2b). Furthermore, we noted that the level of IL-17A (S and MF) were significantly higher in CD103+CD4+T_RM_ than in CD103− CD4+T_RM_ (Fig. 2b). Similar assessments were made for IL-2 and TNFα for background responses (Fig. 2c, d). Interestingly, no differences in either CD4+T_RM_ subset were noted for IL-2 and TNFα, except for CD103− CD4+T_RM_ that showed a decreased trend in IL-2-S (Fig. 2c) and an increased trend in TNFα-S (Fig. 2d) responses following Ty21a-immunization. Interestingly, we also observed significantly higher levels of IL-2-MF and TNFα-MF CD4+CD103+T_RM_ than CD4+CD103−T_RM_ in Ty21a-vaccinees (Fig. 2d).

Likewise, CD4+T_RM_ subsets responses were examined in both groups of volunteers following stimulation with α-CD3/CD28 beads. Remarkably, while α-CD3/CD28 stimulated equally both CD4+T_RM_ subsets to produce higher IFNγ-MF in the two groups, oral Ty21a-immunization significantly induced IFNγ-S responses (Additional file 4: Fig. S4A). When IL-17A responses were examined following stimulation with α-CD3/CD28 beads, no significant differences were observed following Ty21a-immunization (Additional file 4: Fig. S4B). However, CD103+ CD4+T_RM_ produced significantly higher levels of IL-17A-MF than CD103− CD4+T_RM_ (Additional file 4: Fig. S4B). We also determined the IL-2 and TNFα responses of the CD4+T_RM_ subsets following α-CD3/CD28 stimulation (Additional file 4: Fig. S4C, D). Interestingly, while both CD4+T_RM_ subsets produced higher levels of IL-2-MF and TNFα-MF equally in both volunteer groups to α-CD3/CD28 stimulation, IL-2-S was significantly decreased in both subsets following Ty21a-immunization (Additional file 4: Fig. S4C, D). Additionally, CD103+ CD4+T_RM_ produced significantly (p < 0.05) higher levels of TNFα-S and MF than CD103− CD4+T_RM_ following Ty21a-immunization (Additional file 4: Fig. S4D). These granular data suggest that oral Ty21a-immunization influence intrinsic differences of LPMC CD4+T_RM_ resulting in distinct background and stimulatory characteristics.

### LPMC-CD4+T_RM_ subsets respond differently following Ty21a immunization

CD4+T_RM_ respond rapidly following re-exposure to pathogens. However, no information is available on CD4+T_RM_ vaccine-induced responses, particularly the characteristics of CD103+ and CD103− CD4+T_RM_ following oral Ty21a-immunization in the human TI. Thus, we hypothesized that TI CD103+ and CD103− CD4+T_RM_ would contribute differently in the CMI responses following Ty21a immunization.

**Fig. 3:**
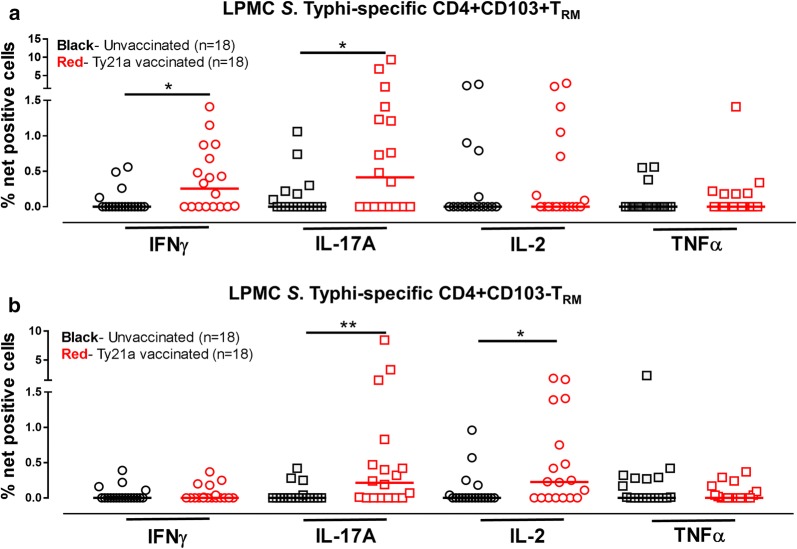
*S.* Typhi-specific responses of terminal ileum LPMC CD4+CD103+ and CD8+CD103− T_RM_ subsets in healthy individuals following oral Ty21a-immunization. The net percentages of *S.* Typhi-specific responses (IFNγ, IL-17A, IL-2, and TNFα) elicited by *S.* Typhi-infected autologous targets in **a** CD4+CD103+ and **b** CD4+CD103− T_RM_ subsets were compared between Ty21a-vaccinated (n = 18; red symbols) and unvaccinated volunteers (n = 18; black symbols) with significant differences (*p < 0.05; **p < 0.005) indicated. Horizontal bars (black and red) represent median values

To test this hypothesis, we evaluated the ability of CD4+T_RM_ subsets to elicit responses following stimulation with autologous *S*. Typhi-infected EBV-B (targets) in Ty21a vaccinated (n = 18) and unvaccinated (n = 18) volunteers. Interestingly, the cumulative data demonstrated that LPMC CD103+ CD4+T_RM_ exhibited significantly (p < 0.05) higher levels of IFNγ and IL-17A in Ty21a-vaccinated than unvaccinated volunteers following Ty21a-immunization (Fig. 3a). No difference in IL-2 and TNFα were noted (Fig. 3a). In contrast, the predominant LPMC CD103− CD4+T_RM_ subset produced significantly (p < 0.05) higher levels of IL-17A and IL-2 following Ty21a-immunization (Fig. 3b). No differences were observed in CD103− CD4+T_RM_ IFNγ and TNFα levels CD103− CD4+T_RM_ (Fig. 3b).

### Multifunctional LPMC CD4+T_RM_ S. Typhi responses following oral Ty21a-immunization

In various diseases including typhoid fever, induction of antigen-specific multifunctional T cells have been shown to be associated with favorable disease outcome, higher effector function and higher protective efficacy after immunization [39–43] compared to monofunctional T cells. In addition, recent evidences show that there are molecular differences between multifunctional and monofunctional CD4^+^ T cells [44]. Thus, we deemed that examination of multifunctional CD4+T_RM_ in the terminal ileum mucosa is important for assessing the quality of the responses following Ty21a immunization.

To further investigate the differences in *S*. Typhi-specific responses between LPMC CD103+ and CD103−CD4+T_RM_, we used the Winlist FCOM function to analyze multiple cytokines (i.e., IFNγ, IL-17A, IL-2, and TNFα) in individual *S*. Typhi-specific responding cells and classified them as either single cytokine producer (S) or multifunctional (Sum of double, triple, and quadruple cytokine producers) (MF).

**Fig. 4:**
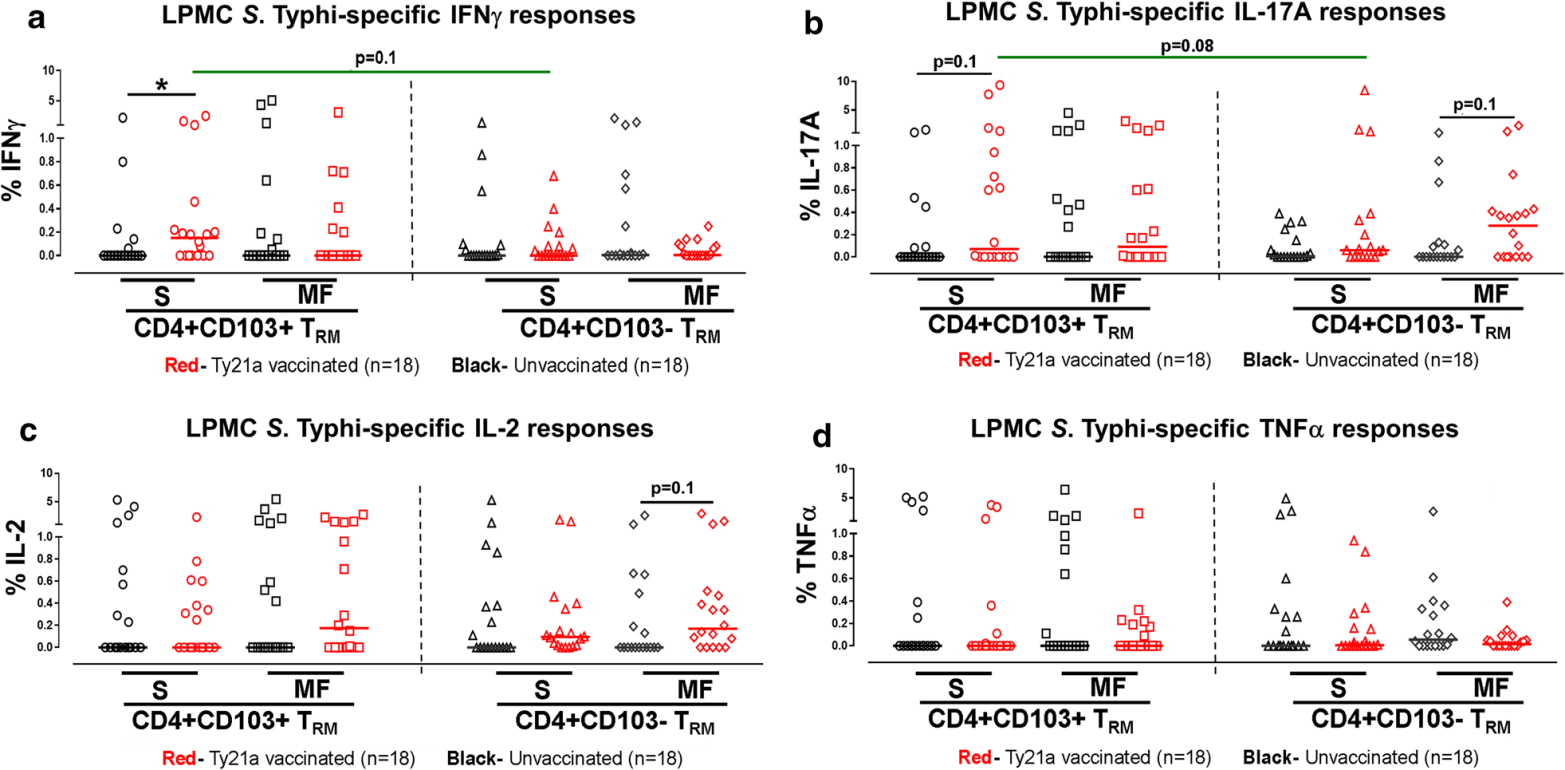
Effect of oral Ty21a immunization on terminal ileum multifunctional and single cytokine-expressing *S.* Typhi-specific CD4+CD103+ and CD4+CD103− T_RM_ cell subsets. Net *S.* Typhi-specific CD4+ T_RM_ subsets responses elicited by *S.* Typhi-infected autologous targets were calculated using the FCOM function of Winlist and stratified into multifunctional cells (MF) and single-positive cells (S). Comparison of TI LPMC CD4+CD103+ and CD4+CD103− T_RM_*S.* Typhi-specific **a** INFγ+; **b** IL-17A+; **c** IL-2+, and **d** TNFα+ MF and S in Ty21a-vaccinated (n = 18; red symbols) and unvaccinated volunteers (n = 18; black symbols). Significant differences are shown (*p < 0.05). Black lines: differences between Ty21a vaccinated and unvaccinated volunteers. Green lines: differences between CD4+CD103+ and CD4+CD103− T_RM_ responses. Trends are represented by their p-values. Horizontal bars (black and red) represent median values

First, we analyzed the net *S*. Typhi-specific IFN-γ responses of LPMC CD4+T_RM_ (CD103+ and CD103−) subsets stimulated with autologous targets for multi-functionality in Ty21a-vaccinees and controls (Fig. 4). Interestingly, no significant differences were noted in LPMC CD103− CD4+T_RM_ (S or MF) IFN-γ responses following Ty21a-immunization (Fig. 4a). In contrast, CD103+ CD4+T_RM_ produced significantly higher level of IFNγ-S but not MF following Ty21a-immunization (Fig. 4a). Both LPMC CD4+T_RM_ subsets exhibited higher levels of IL-17A following Ty21a-immunization (Fig. 4b). However, CD103+ CD4+T_RM_ produced higher levels of IL-17A-S (p = 0.1) whereas CD103− CD4+T_RM_ exhibited increases in IL-17A-MF (p = 0.1) (Fig. 4b). Furthermore, CD103+CD4+T_RM_ showed a trend for higher levels of IL-17A-S production than CD103− CD4+T_RM_ (green line) (Fig. 4b). Similarly, we determined MF IL-2 and TNFα responses of CD103+ and CD103−CD4+T_RM_. No significant differences were observed in IL-2-S and TNFα-S or MF responses from CD4+T_RM_ subsets following Ty21a-immunization (Fig. 4c, d). However, CD103− CD4+T_RM_ exhibited a trend (p = 0.1) to higher responses of IL-2-MF following Ty21a-immunization (Fig. 4c).

### LPMC-CD4+T_RM_ subsets exhibited distinct responses to stimulation with a Ty21a homogenate antigen preparation

CD4+T_M_ cells have been shown to respond to both autologous *S*. Typhi-infected and soluble antigens (e.g., Ty21a homogenate) [35]. Thus, we stimulated both CD4+T_RM_ subsets with Ty21a homogenate (10 μg/mL) and determined their net responses (% of Ty21a homogenate responses—% of unstimulated (cell alone) responses) in a subgroup of Ty21a vaccinated (n = 8) and unvaccinated (n = 8) volunteers. Remarkably, following stimulation with Ty21a homogenates, no significant differences were noted in cytokines (IFNγ, IL-17A, IL-2 and TNFα) producing CD103+ CD4+T_RM_ between the volunteer groups (Additional file 5: Fig. S5A). In contrast, CD103− CD4+T_RM_ elicited significantly higher levels of IFNγ and TNFα in Ty21a vaccinated than unvaccinated volunteers following stimulation with Ty21a homogenate (Additional file 5: Fig. S5B). Taken together, these data suggest that CD4+T_RM_ subsets exhibit unique characteristics following Ty21a immunization.

**Fig. 5:**
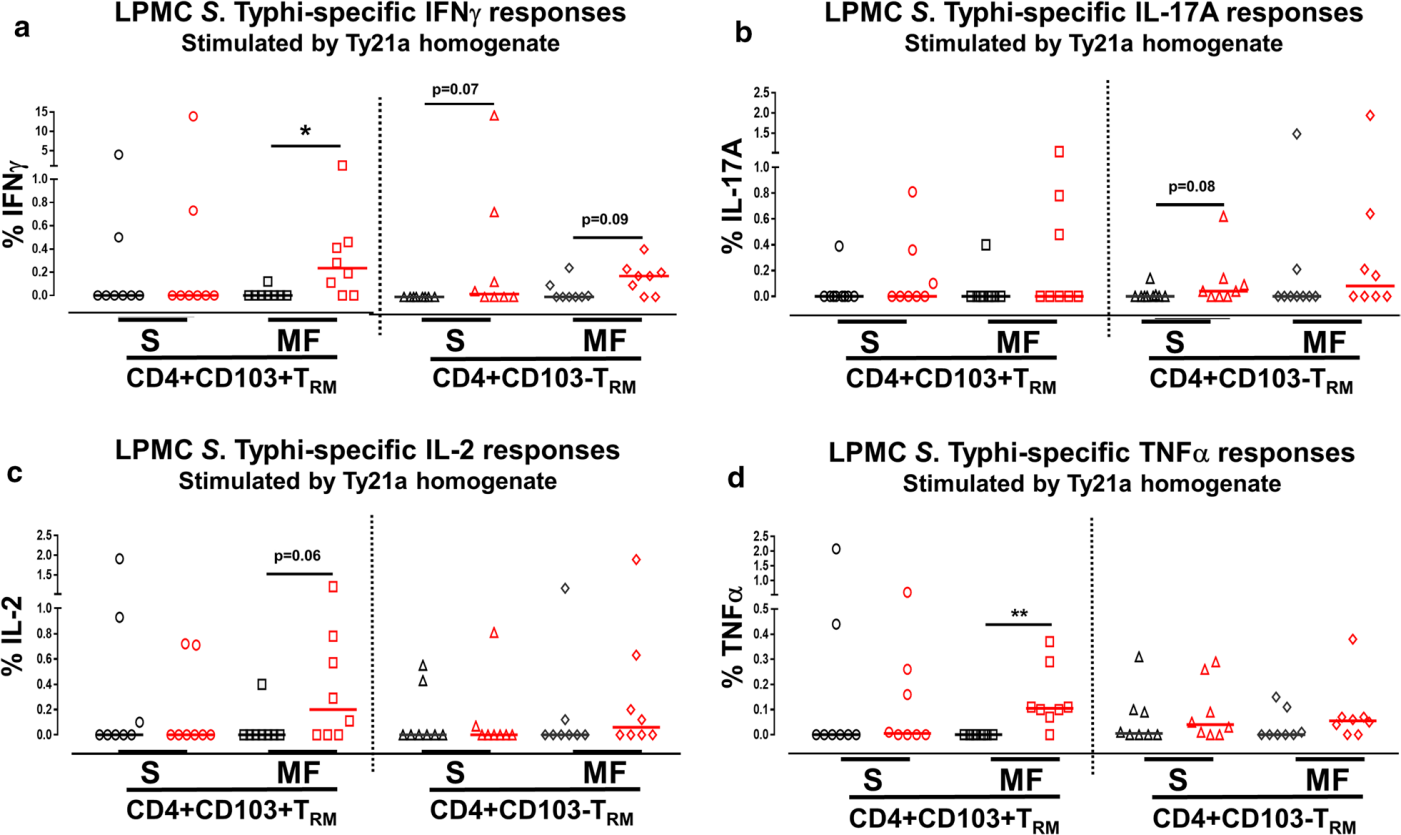
Multifunctional *S.* Typhi-specific responses to a Ty21a homogenate antigenic preparation by terminal ileum LPMC CD4+CD103+ and CD4+CD103− T_RM_ subsets in healthy adults following oral Ty21a immunization. Terminal ileum LPMC CD4+CD103+ and CD4+CD103− T_RM_ were stimulated with Ty21a homogenate antigen (10 μg/mL) as described in Materials and Methods. Net Ty21a homogenate (media subtracted) *S.* Typhi-specific CD4+ T_RM_ subsets responses were calculated using the FCOM function of Winlist and stratified into multifunctional cells (MF) and single-positive cells (S). Comparison of TI LPMC CD4+CD103+ and CD4+CD103− T_RM_ Ty21a homogenate mediated *S.* Typhi-specific **a** INFγ+; **b** IL-17A+; **c** IL-2+, and **d** TNFα+ MF and S responses in Ty21a-vaccinated (n = 8; red symbols) and unvaccinated volunteers (n = 8; black symbols). Significant differences are shown (*p < 0.05; **p < 0.005). Trends are represented by their p-values. Horizontal bars (black and red) represent median values

Next, we studied *S*. Typhi-specific IFN-γ multifunctional responses following Ty21a homogenate stimulation in CD103+ and CD103− CD4+T_RM_ obtained from Ty21a-vaccinated and unvaccinated volunteers (Fig. 5). CD103+ CD4+T_RM_ produced significantly higher levels of IFNγ-MF but not S in Ty21a-vaccinees than controls (Fig. 5a). However, CD103− CD4+T_RM_ only showed trends to exhibit increases of IFNγ-S and MF in Ty21a-vaccinated compared to unvaccinated volunteers following stimulation with the Ty21a homogenate (Fig. 5a). Likewise, we determined multifunctional IL-17A responses and observed no significant differences in IL-17A-S or MF production following Ty21a-immunization (Fig. 5b) except for CD103− CD4+T_RM_ that exhibited a trend to show increased responses by IL-17A-S in Ty21a-vaccinees (Fig. 5b). Similarly, we determined IL-2 and TNFα multifunctional responses from CD4+T_RM_ subsets. No significant differences in IL-2-S or MF and TNFα-S or MF CD103− CD4+T_RM_ responses were found following Ty21a-immunization (Fig. 5c, d). However, CD103+ CD4+T_RM_ exhibited significantly higher levels of TNFα-MF and a trend to show increased IL-2-MF following Ty21a-immunization (Fig. 5c, d).

### Oral Ty21a-immunization elicits *S*. Typhi-specific terminal ileum IEL CD4+T_RM_

**Fig. 6:**
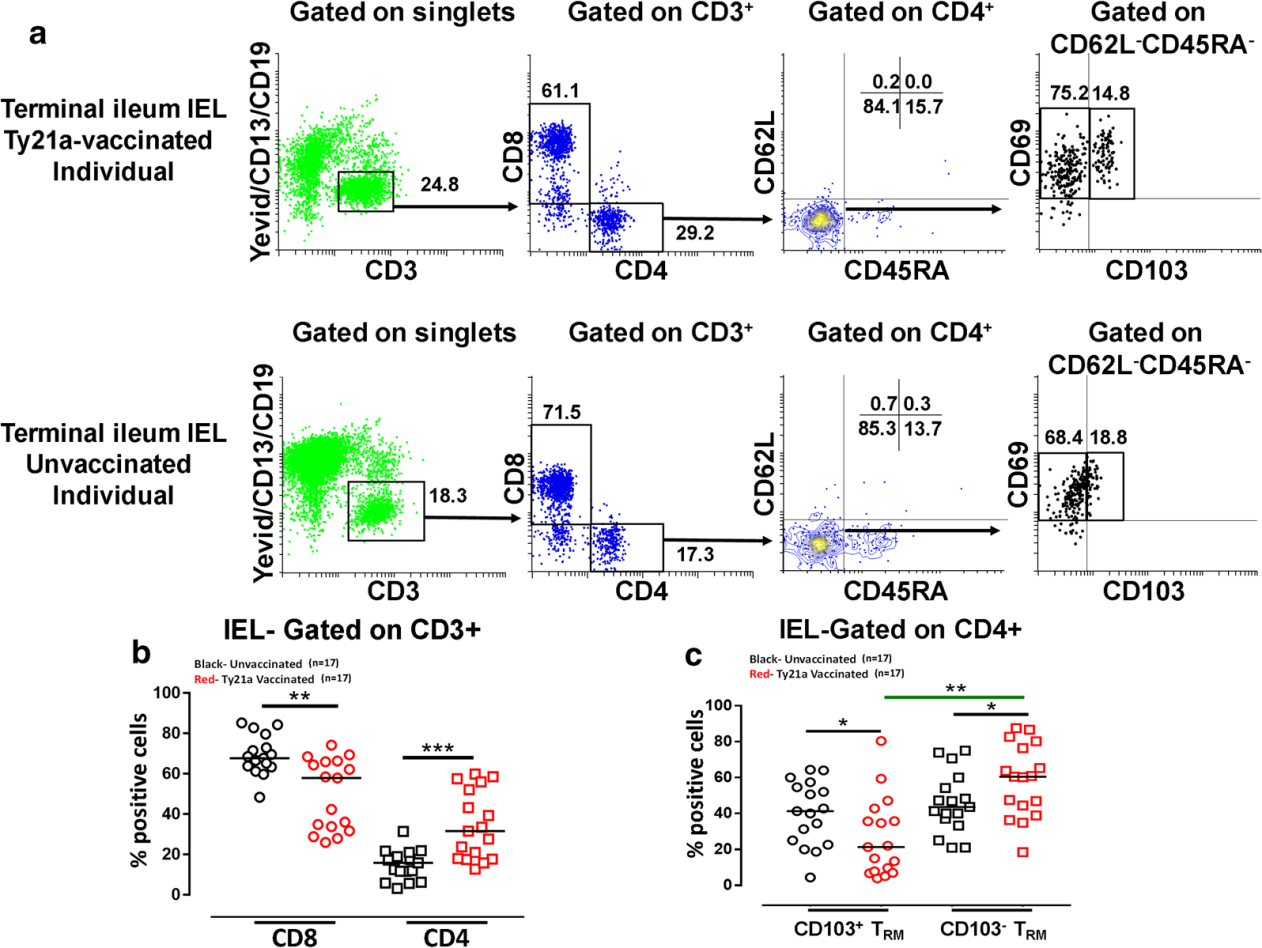
Gating Strategy for identifying terminal ileum intraepithelial T lymphocytes (IEL)-CD4+ tissue-resident T memory cell subsets (T_RM_) and impact of oral Ty21a-immunization on IEL CD4+ T_RM_ subsets frequencies. Terminal ileum intraepithelial lymphocytes (IEL) were isolated and IEL CD4+ T_RM_ studied in Ty21a vaccinated, and unvaccinated individuals using a combination of CD69 and CD103 markers. **a** Representative gating strategies for a Ty21a vaccinated and an unvaccinated participant are shown. **b** The frequencies of terminal ileum IEL CD4+ T and CD8+ T cells were determined and compared between TI IEL obtained from Ty21a-vaccinated (n = 17; red symbols) and unvaccinated volunteers (n = 17; black symbols). **c** The frequencies of terminal ileum IEL CD4+CD103+ and CD4+CD103− T_RM_ cells were determined and compared between TI IEL obtained from Ty21a-vaccinated (n = 17; red symbols) and unvaccinated volunteers (n = 17; black symbols). Significant differences are shown (*p < 0.05; **p < 0.005; ***p < 0.0005). Horizontal bars represent median values

The intestinal mucosa is composed of a single layer of intestinal epithelial cells that is superimposed on the LP. Because LPMC CD103 +CD4+T_RM_ expresses CD103, a ligand for E-cadherin on epithelial cells, it is reasonable to speculate that CD103+ CD4+T_RM_ are poised to migrate and contribute to *S*. Typhi responses in the epithelial microenvironment. Thus, we hypothesized that oral Ty21a-immunization might influence the frequencies and baseline responses of intraepithelial lymphocytes (IEL) CD4+T_RM_ in the human TI epithelial compartment. To address this hypothesis, we freshly isolated IEL and phenotypically characterized CD4+, CD8+ T cells and CD4+T_RM_ subsets in Ty21a-vaccinated (n = 17) and unvaccinated (n = 17) volunteers. Representative examples of the gating strategy for a Ty21a vaccinated and an unvaccinated individual are shown in Fig. 6a.

Interestingly, we observed that the frequencies of IEL-T cells were significantly altered following oral Ty21a immunization. Specifically, the frequencies of CD3+CD8+ IEL T cells were significantly (p < 0.005) decreased while CD3+CD4+ IEL T cell frequencies were significantly (p < 0.0005) increased following Ty21a-immunization (Fig. 6b). To confirm that there were no discrepancies in cell yields, the number of viable IEL per mg of TI tissues obtained from biopsies of Ty21a-vaccinated and unvaccinated volunteers were compared. No significant differences in cell yields were observed between the two groups (Additional file 6: Fig. S6). Similarly, almost identical cell yields were observed in LPMC between the two groups. However, significantly higher cell numbers were observed in LPMC as compared to IEL (Additional file 6: Fig. S6).

Next, we observed that TI CD103^+^ and CD103− CD4+T_RM_ were present in similar proportions in IEL as in LPMC (Fig. 6a). Interestingly, the frequencies of IEL CD103+ CD4+T_RM_ were significant (p < 0.05) decreased following oral Ty21a-immunization (Fig. 6C). In contrast, IEL CD103− CD4+T_RM_ frequencies were significantly (p < 0.05) increased in Ty21a vaccinated as compared to unvaccinated volunteers (Fig. 6c). Thus, these data suggest that the increase in IEL CD4+T cells result mainly from increases in the CD103− CD4+T_RM_ subset.

**Fig. 7:**
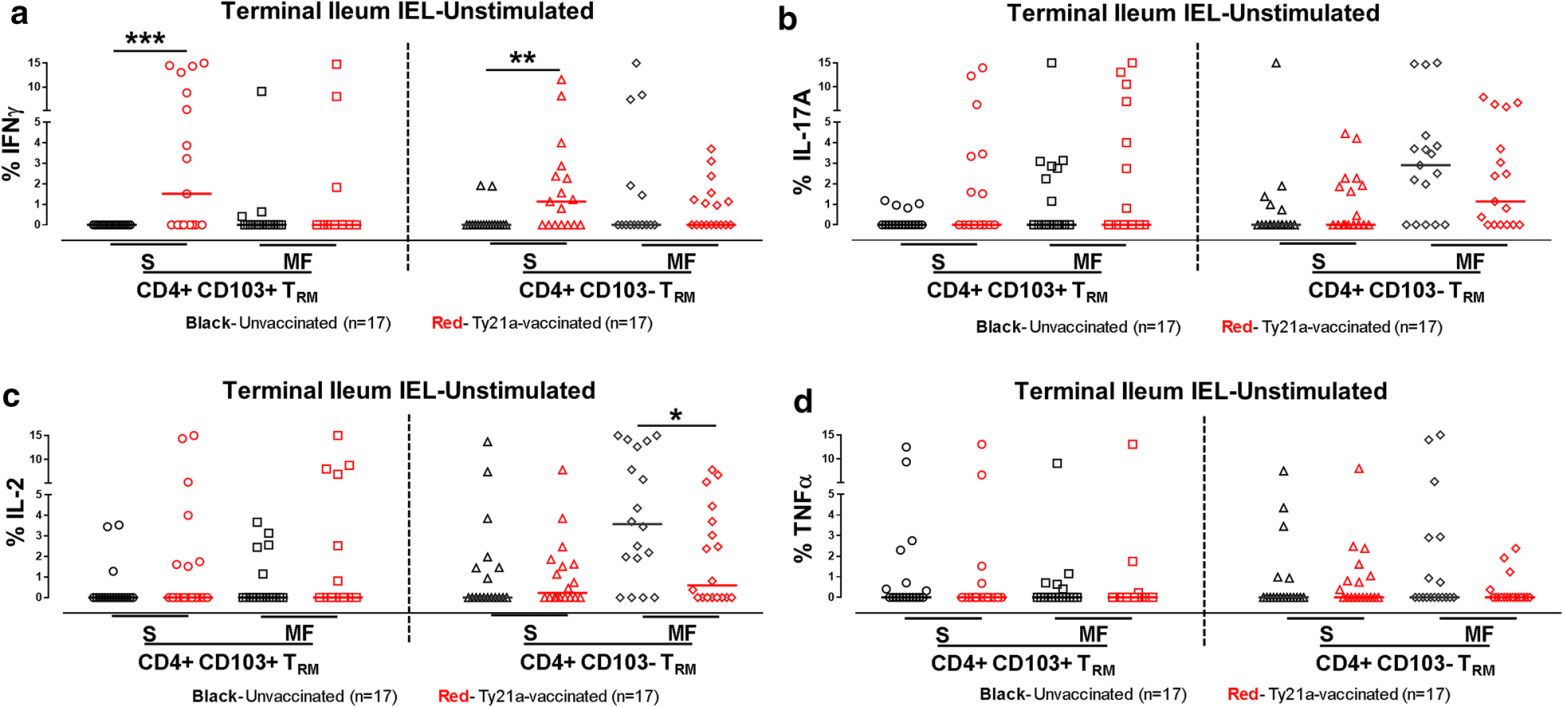
Effect of oral Ty21a immunization on ex-vivo terminal ileum IEL CD4+CD103+ and CD8+CD103− T_RM_. Ex-vivo unstimulated IEL CD4+CD103+ and CD4+CD103− T_RM_ were cultured overnight and their spontaneous cytokine (IFNγ, IL-17A, IL-2 and TNFα) production were measured using flow cytometry. Using the FCOM function of WinList, IEL CD4+CD103+ and CD4+CD103− T_RM_ responses were stratified into single-positive cells (S) and multifunctional cells (MF). Comparisons of TI IEL CD4+CD103+ and CD4+CD103− T_RM_ responses in **a** INFγ+; **b** IL-17A+; **c** IL-2+, and **d** TNFα+ S and MF in Ty21a-vaccinated (n = 17; red symbols) and unvaccinated volunteers (n = 17; black symbols) with significant differences shown (*p < 0.05; **p < 0.005; ***p < 0.0005). Black lines: significant differences between Ty21a vaccinated and unvaccinated volunteers. Horizontal bars (black and red) represent median values

We next measured and compared the frequency of baseline *S*. Typhi single (S) and multifunctional (MF) producing cytokines (IFNγ, IL-17A, IL-2 and TNFα) in unstimulated IEL-CD4+T_RM_ subsets obtained from Ty21a vaccinated (n = 17) and unvaccinated (n = 17) volunteers following an overnight incubation (Fig. 7). Ex-vivo unstimulated IEL CD4+T_RM_ obtained from Ty21a immunized volunteers exhibited significantly higher level of IFNγ-S but not MF than controls (Fig. 7a). In contrast, no significant differences in the production of spontaneous IL-17A-S or MF, TNFα-S or MF and IL-2-S from IEL CD4+T_RM_ subsets following Ty21a-immunization (Fig. 7b–d). Of note, IEL CD103− CD4+T_RM_ produced significantly (p < 0.05) less IL-2-MF in Ty21a vaccinated than in unvaccinated volunteers (Fig. 7c). We then studied multifunctional responses of IEL CD103+ and CD103− CD4+T_RM_ in Ty21a vaccinated (n = 10) and unvaccinated (n = 8) volunteers following stimulation with α-CD3/CD28 beads. Remarkably, while α-CD3/CD28 stimulated equally CD4+T_RM_ subsets from both groups (Additional file 7: Fig. S7A–D), we observed trends to show significant increases in TNFα-S CD103+ CD4+T_RM_ and in IFNγ-S CD103−CD4+T_RM_, as well as significant (p < 0.05) increases in TNFα-S responses by CD103− CD4+T_RM_ in Ty21a vaccinees (Additional file 7: Fig. S7A–D).

### Oral Ty21a-immunization elicits S. Typhi responsive CD4+T_RM_ in the intestinal epithelial compartment

Above we showed that LPMC *S*. Typhi-specific CD4+T_RM_ were elicited by oral Ty21a immunization. Thus, we hypothesized that oral Ty21a-immunization would induce distinct *S*. Typhi specific IEL CD4+T_RM_ responses. To test this hypothesis, net *S*. Typhi responsive IEL CD4+T_RM_ were evaluated following stimulation with autologous *S*. Typhi-infected targets in Ty21a vaccinated (n = 7) and unvaccinated (n = 6) volunteers. No significant differences were observed in cytokine production (IFNγ, IL-17A, IL-2 and TNFα) from IEL CD103+ CD4+T_RM_ following Ty21a-immunization (Additional file 8: Fig. S8A). In contrast, the predominant IEL CD103− CD4+T_RM_ exhibited a trend to show increased IFNγ and TNFα (p = 0.06) responses in Ty21a-vaccinated than in unvaccinated volunteers (Additional file 8: Fig. S8B).

**Fig. 8:**
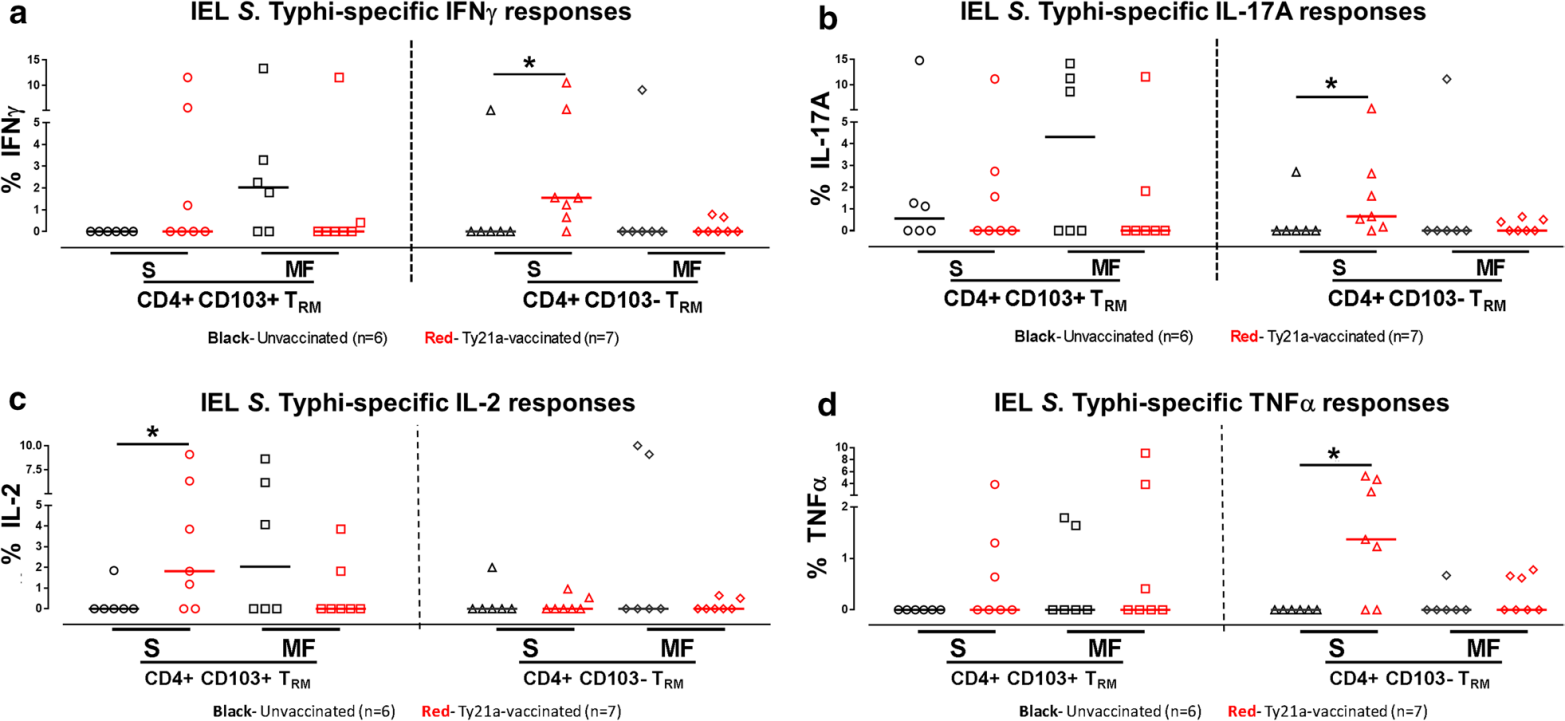
Effect of oral Ty21a immunization on terminal ileum multifunctional and single cytokine expressing *S.* Typhi-specific IEL CD4+CD103+ and CD4+CD103− T_RM_ cell subsets. Net *S.* Typhi-specific IEL CD4+ T_RM_ subsets responses elicited by *S.* Typhi-infected autologous targets were calculated using the FCOM function of WinList and stratified into multifunctional cells (MF) and single-positive cells (S). Comparison of TI IEL CD4+CD103+ and CD4+CD103− T_RM_*S.* Typhi-specific **a** INFγ+; **b** IL-17A+; **c** IL-2+, and **d** TNFα+ MF and S in Ty21a-vaccinated (n = 7; red symbols) and unvaccinated volunteers (n = 6; black symbols). Significant differences are shown (*p < 0.05). Horizontal bars (black and red) represent median values

Next, we studied multifunctional *S.* Typhi-specific IEL-CD4+ T_RM_ responses in Ty21a-vaccinated (n = 7) and unvaccinated (n = 6) volunteers. First, we evaluated IFN-γ responses in IEL CD103+ and CD103− CD4+T_RM_ and observed no significant differences in IEL-CD103+ CD4+T_RM_ (S or MF) responses following Ty21a-immunization (Fig. 8a). In contrast, CD103− CD4+T_RM_ showed significant (p < 0.05) increases in IFNγ-S but not MF in Ty21a-vaccinated than unvaccinated volunteers (Fig. 8a). Next, we determined multifunctional IL-17A responses from IEL CD4+T_RM_ subsets. No significant differences were detected in IEL CD103+ CD4+T_RM_ (S or MF) responses following Ty21a-immunization (Fig. 8b). However, IEL-CD103− CD4+T_RM_ produced significantly (p < 0.05) higher level of IL-17A following Ty21a-immunization (Fig. 8b). Similarly, we determined multifunctional IL-2 responses from IEL CD4+T_RM_ subsets. No significant differences were observed in IEL CD103− CD4+T_RM_ (S or MF) responses following Ty21a-immunization (Fig. 8c). However, CD103+ CD4+T_RM_ exhibited significant increases in IL2-S but not MF in Ty21a-vaccinated compared to unvaccinated volunteers (Fig. 8c). Finally, we assessed multifunctional TNFα responses and found no significant differences in IEL CD103+ CD4+T_RM_ (S or MF) responses following Ty21a-immunization (Fig. 8d). However, IEL CD103- CD4+T_RM_ produced significantly (p < 0.05) higher levels of TNFα-S but not MF in Ty21a-vaccinated compared to unvaccinated volunteers (Fig. 8d). We conclude that oral Ty21a-immunization elicits mainly cytokine-producing IEL CD103− CD4+T_RM_ in the TI epithelium.

Intestinal T_RM_ (CD4+ and CD8+) are located in both the lamina propria and epithelial compartments, which provides unique immunological niches. Thus, we hypothesized that the CD69 and CD103 expression profile of CD4+T_RM_ subsets may differ between LP and epithelial compartments following Ty21a immunization. To address this hypothesis, we measured the mean fluorescence intensity (MFI) of CD69 and CD103 expressed on LPMC and IEL CD4+T_RM_ subsets obtained simultaneously from Ty21a vaccinated (n = 8) and unvaccinated (n = 8) volunteers (Additional file 9: Fig. S9). As shown in cytograms from a representative unvaccinated volunteer, CD103 is highly expressed on both LPMC and IEL CD103+ CD4+T_RM_ with similar intensities in IEL and LPMC (Additional file 9: Fig. S9A). Next, we compared the level of CD103 MFI between IEL and LPMC CD4+T_RM_ subsets obtained from either Ty21a-vaccinated (n = 8) or unvaccinated (n = 8). Cumulative data showed no significant differences in the levels of CD103 expression between LPMC and IEL CD4+T_RM_ subsets (Additional file 9: Fig. S9B).

We also compared the levels of CD69 expression (MFI) of CD4+T_RM_ subsets between LPMC and IEL following Ty21a-immunization as shown in an unvaccinated representative volunteer (Additional file 9: Fig. S9C). Interestingly, significantly (p < 0.005) higher expression of CD69 was detected in LPMC CD103+ CD4+T_RM_ than in LPMC CD103− CD4+T_RM_ in unvaccinated volunteers (Additional file 9: Fig. S9D). Similarly, we observed a trend (p = 0.07) to show higher levels of CD69 expression in LPMC CD103+ CD4+T_RM_ than in LPMC CD103− CD4+T_RM_ following Ty21a-immunization (Additional file 9: Fig. S9D). However, there were no significant differences in CD69 MFI levels in Ty21a-vaccinated compared to unvaccinated volunteers in either LPMC CD4+T_RM_ subsets (Additional file 9: Fig. S9D).

In contrast in IEL we observed significantly (p < 0.005) higher expression of CD69 in IEL-CD103+ CD4+T_RM_ than IEL CD103− CD4+T_RM_ in Ty21-vaccinated volunteers (Additional file 9: Fig. S9D). No differences in CD69 expression were observed between CD4+T_RM_ subsets in unvaccinated volunteers (Additional file 8: Fig. S8D). However, significant (p < 0.05) decreases in CD69 expression were observed in IEL CD103− CD4+T_RM_ following Ty21a-immunization (Fig. S9D). Comparisons between the two compartments showed trends for CD69 expression on CD103+ CD4+T_RM_ to display higher levels of CD69 expression (p = 0.10) in IEL than LPMC following Ty21a-immunization (Additional file 8: Fig. S8D). In contrast, a trend (p = 0.10) to show increases in CD69 expression was noted in CD103− CD4+T_RM_ in IEL compared to LPMC from unvaccinated volunteers (Additional file 9: Fig. S9D).

Furthermore, we explored the relationship between the generation of *S*. Typhi specific immune responses between LPMC and IEL individually by performing Spearman correlation tests between LPMC and IEL CD4+T_RM_ S and MF responses in both Ty21a vaccinated and unvaccinated volunteers. Interestingly, in unvaccinated volunteers, the frequencies of LPMC CD103+CD4+ T_RM_ IL-2-S responses only were significantly positively correlated to their IEL counterparts while CD103−CD4+T _RM_ subset has significant positive correlation only in IL-17A-S (Additional file 10: Table S1). However, following Ty21a-vaccination, the frequencies of LPMC CD103+CD4+ T_RM_ S (IFNγ) were significantly positive correlated while their MF (IL-17A and IL-2) counterparts were significantly negatively correlated to their IEL counterparts (Additional file 10: Table S1). In contrast, the frequencies of LPMC CD103−CD4+T_RM_ S (IFNγ and IL-17A-negative; TNFα-positive) but not MF were significantly correlated to their IEL counterparts (Additional file 10: Table S1).

## Discussion

Vaccine-mediated protective immunity is generally enabled by the induction of both, antibodies and appropriate cellular immune responses, including those mediated by CD4+T cells. There is growing evidence that T_RM_ (e.g., CD4+T_RM_) cells play a crucial role in protective immunity following natural infection and their subsequent secondary exposure. However, there are no previous reports on the role of CD4+T_RM_ following oral Ty21a-immunization in the human TI. Here, we examined the responses elicited by Ty21a-immunization on TI LPMC and IEL CD4+T_RM_ subsets. We observed that Ty21a-immunization activated LPMC CD103+ and CD103− CD4+T_RM_ resulting in increased spontaneous cytokines (IFNγ, TNFα and IL-17A) production. Importantly, LPMC CD4+T_RM_ subsets contributed significantly to *S*. Typhi-specific IFNγ, IL-17A and IL-2 responses following stimulation with *S*. Typhi-infected targets. Moreover, these responses differed in magnitude and characteristics between CD103+ and CD103− CD4+T_RM_, suggesting a dichotomy in their contributions and possibly different roles in *S*. Typhi immunity. Finally, we demonstrated that Ty21a-immunization induces IEL CD4+T_RM_ subsets in the epithelial compartment both spontaneously and following antigen-specific stimulation. Interestingly, IEL CD103− CD4+ T_RM_ contributed significantly to *S*. Typhi specific IFNγ, IL-17A and TNFα as single producing cells while IEL CD103+CD4+T_RM_ contributed to IL-2 production. Taken together, these results contribute major novel information of the effects of oral Ty21a-vaccination and the role of CD4+T_RM_ in human TI mucosal responses.

The intestinal mucosa is a major site of antigenic exposure from various sources including the microbiome and enteric pathogens. Multiple specialized populations of immune cells (adaptive and innate) including CD4+T_RM_ reside in the intestine and contribute to intestinal “homeostasis” and protective immunity against enteric pathogens. *S*. Typhi or the attenuated *S.* Typhi vaccine Ty21a strain can effectively invade the TI mucosa, preferred site of *S.* Typhi infection [45, 46]. Then, it is reasonable to hypothesize that exposure to *S.* Typhi elicits both innate and adaptive responses including CD4+T_RM_ in the lamina propria and the epithelial compartments of the TI which might play a significant role in protection against *S*. Typhi. The complexities involved in obtaining cells from the human terminal ileum biopsies (LPMC and IEL), are likely to have contributed to the lack of information available on CD4+T_RM_ immune responses following oral vaccination. Using this unique model of oral live attenuated Ty21a vaccine in humans, we showed that oral Ty21a-immunization resulted in the spontaneous production of cytokine cells in the LP from CD103+ (IFNγ-S and IL-17A-S) and CD103− (IFNγ-S and TNFα-S) CD4+T_RM_ and in the epithelium where IFNγ-S was produced spontaneously from both CD4+T_RM_ subsets. Similar effects (e.g., increase in IFNγ-S and TNFα-S in both LP and IEL) elicited by Ty21a-immunization were observed following stimulation with α-CD3/CD28 stimulation. We hypothesize that there may be multiple cell subsets within each CD4+T_RM_ subpopulation that differ in their requirements for activation and/or their recognition of specific cognate antigens [47–49]. Future studies should focus on fully understanding the heterogeneity and contribution of the resident cells in spontaneous cytokine production following Ty21a-immunization. These cells might play a role in protection, albeit likely to be of lesser importance than those mediated by antigen-specific responses. Overall, these unique data indicate that immunization with bacterial oral vaccines may have immunomodulatory effects beyond those that are specific for the vaccine being administered leading to a generalized level of activation.

While the cellular requirements and transcriptional basis for the induction of CD8+T_RM_ has been studied in other systems, our understanding of CD4+T_RM_ generation and maintenance is poor. CD4+T_RM_ unlike CD8+T_RM_ are less uniform in their expression of CD103 and consequently two distinct subsets are present in tissues. Here, TI CD103+ and CD103− CD4+T_RM_ elicited immune responses were evaluated following Ty21a immunization. Our data provided the first evidence of distinct responses between these two subsets in terms of spontaneous secretion of cytokines, stimulation with anti-CD3/CD28, and *S*. Typhi-specific responses resulting from stimulation with *S*. Typhi-infected targets and Ty21a homogenate antigens. Specifically, our results showed striking differences in terms of the characteristics (IFNγ and S for CD103+; IL-2 and MF for CD103−) of these responses. Of note, a recent study reported differences in IFNγ responses between lung CD103+ and CD103− CD4+T_RM_ following stimulation with α-CD3/CD28 [7]. Taken together, these results suggest that CD103+CD4+T_RM_ subset (Th1 and Th17) are distinct from CD103− CD4+T_RM_ subset (Th1) and might play a different role in the mucosa following oral vaccination and/or infection.

We have previously characterized *S*. Typhi-specific LPMC CD4+T_EM_ cells and observed increasing trends in IFNγ and IL-17A production following Ty21a immunization [50]. Here, we showed that the IFNγ and IL-17A are primarily produced by CD4+T_RM_ (a subset of CD4+ T_EM_) in significantly higher levels in the terminal ileum mucosa following Ty21a immunization. In addition, we observed that CD103+ CD4+T_RM_ produced cytokines as monofunctional cells whereas CD103− CD4+T_RM_ cells produced mostly multifunctional cytokines, similar to our observations in LPMC CD4+T_EM_ [50]. These results suggest that examination of heterogeneous CD4+ T populations may mask the ‘true’ impact of oral Ty21a immunization due to the ‘averaging effect’ inherent to the analyses of whole populations/subsets which are composed of responding and non-responding cells. Thus, it is essential to focus on the fine granularity (e.g., *S.* Typhi-specific CD103− or CD103+ T_RM_ subsets, S vs MF) to better characterize the responses and properly study differences between cells in various immune compartments. Here, we demonstrated that oral Ty21a immunization elicited CD4+ T_RM_ subsets in both, the LP and epithelial compartments, involving effector mechanisms (e.g., IL-17A) that might be well suited for protection against intracellular pathogens.

The type of antigen used for in vitro stimulation influence the responses of CD4+T_M_. Thus, we addressed this issue by using *S*. Typhi-infected targets (moderately efficient CD4+ T stimulation) and Ty21a homogenate antigens (an efficient CD4+ T stimulation) [51] to stimulate CD4+T_RM_ subsets. Remarkably, our results indicate that following stimulation with Ty21a homogenate antigens, LPMC CD103+T_RM_ largely produced significantly higher levels of *S*. Typhi-specific IFNγ-MF, IL-2-MF and TNFα-MF in Ty21a vaccinated participants than those in the unvaccinated group. These results were different from those observed following stimulation with *S*. Typhi-infected targets (IFNγ-S and IL-17A-S), which largely display *S*. Typhi antigens in the context of both MHC-I and II molecules. Taken together, these data suggest that LPMC CD4+T_RM_ subsets are versatile in responding to *S*. Typhi antigens. Of note, we recently described that oral Ty21a-immunization elicits the induction of *S*. Typhi-specific CD8+T_RM_ [16] and were able to respond following in vitro stimulation with soluble antigens and *S*. Typhi-infected targets, indicating that multiple effector T cell responses are likely to be concomitantly induced. Future studies focusing on the activation requirements of CD4 and CD8 populations in human tissues would be vital to better understand these phenomena to accelerate the development of mucosal vaccines targeting the induction of CMI.

Intestinal T cells are located diffusely throughout the epithelial compartment as intraepithelial lymphocytes (IEL). The predominant population of IEL-T cells are CD8+T with a minor population of CD4+T cells. IEL are part of the first line of defense and are deemed to be an important cell subset involved in immune responses at mucosal surfaces. Thus, in this study, we hypothesized that CD103+CD4+T_RM_ (CD103 binds to E-cadherin on IEC) located in the LP are poised to migrate to the epithelium and play a consequential role in *S*. Typhi immunity. We describe, for the first time, that oral Ty21a-immunization influenced the frequencies of IEL-T cells with a significant shift in the proportion of CD4+T to CD8+T cells (significantly lower CD8+ and higher CD4+T cells). Surprisingly, we observed that this increase in frequency of IEL CD4+T cells was chiefly in the IEL CD103− CD4+T_RM_ subset but not in the CD103+CD4+T_RM_ subset. These data suggest that there may be other receptor-ligand pair(s) (apart from CD103/E-cadherin) involved in the migration of CD103− CD4+T_RM_ from LP to the epithelial compartment. Alternatively, CD103− CD4+T_RM_ cells may be more likely to move freely around the epithelium since it does not depend on their interaction with the E-cadherin on epithelial cells. Regarding *S.* Typhi-specific cytokine production, we found that oral Ty21a-immunization elicits significant IEL CD103+ CD4+ T_RM_ mainly producing IL-2-S. In contrast, IEL CD103− CD4+T_RM_ exhibited significant increases in IFNγS, IL-17A-S and TNFα-S following stimulation with *S.* Typhi-infected targets in Ty21a-vaccinated than unvaccinated volunteers. These antigen-specific responses showed diverse characteristics in the epithelium, suggesting that migrating and/or locally differentiated CD103− CD4+T_RM_ are likely Th1 and Th17 which might play a significant role in *S*. Typhi protective immunity. In contrast, CD103+ CD4+T_RM_ producing IL-2 might be important in supporting T cells proliferation and likely supporting T regulatory cells in this compartment. Taken together, these data suggest that following oral Ty21a immunization, both CD4+T_RM_ effector subsets are recruited from the LP and activated/differentiated uniquely in the epithelium, contributing distinct effector responses involved in effective *S*. Typhi immunity. This compartmentalization of T_RM_ responses in the TI LPMC and IEL may provide important clues for the optimal design of future vaccines.

## Conclusion

In summary, we provide the first evidence of the induction of increased spontaneous and *S.* Typhi-specific cytokine production by CD4^+^ T_RM_ in the human TI LP and epithelial compartments following oral Ty21a-immunization. These results contribute novel insights in our understanding of the generation of gut local immunity in humans following immunization with oral attenuated bacteria and suggest that CD4^+^ T_RM_ play a key role in protection following immunization and/or infection with *S.* Typhi.

## Supplementary information

**Additional file 1: Figure S1.** Study design. Oral typhoid vaccine Ty21a dose schedule (4 doses at -21 to -14 days) and time of collection of specimens (blood and terminal ileum (TI) biopsies) from volunteers undergoing routine screening colonoscopies. Autologous EBV-B cells were generated from pre-immunization blood.

**Additional file 2: Figure S2.** Gating Strategy for the measurement of CD4+ T_RM_ cells in PBMC. PBMC were stained for T memory cell (T_M_) cell subsets, as well as CD69 and CD103 markers, and analyzed following the gating strategy shown.

**Additional file 3: Figure S3.** Activation of LPMC CD4+ T_RM_ cell subsets isolated from terminal ileum of a Ty21a-vaccinated representative volunteer. (A) CD4+CD69+CD103+ T_RM_ and (B) CD4+CD69+CD103− T cells were stimulated with non-infected or *S*. Typhi-infected autologous EBV-B cells and produced cytokines (IFNγ, IL-17A, IL-2 and TNFα) evaluated. Anti (α)-CD3/CD28 stimulation was used as a positive control in both subsets while unstimulated LPMC CD4+CD69+CD103+ or CD4+CD69+CD103− T_RM_ alone (unstimulated) were used as negative controls. The percentage of positive cells in the gated regions is shown above the corresponding black boxes. Net % increases in *S.* Typhi responses (*S.* Typhi-infected EBV-B minus non-infected EBV-B) are shown in the boxes below.

**Additional file 4: Figure S4.** Oral Ty21a-immunization induces differential activation on terminal ileum LPMC CD4+ T_RM_ subsets single cytokine responses following anti-CD3/CD28 stimulation. Following anti-CD3/CD28 stimulation, CD4+CD103+ and CD4+CD103− T_RM_ subset cytokine responses were stratified into multifunctional (**MF**) and single-positive effectors (S). Comparison of TI LPMC CD4+CD103+ and CD4+CD103− T_RM_ subsets responses in (A) INFγ+; (B) IL-17A+; (C) IL-2+, and (D) TNFα+ MF and S in Ty21a-vaccinated (n=18; red symbols) and unvaccinated volunteers (n=18; black symbols) were determined with significant differences shown (*p < 0.05; **p < 0.005; ***p < 0.0005). Black lines: significant differences between Ty21a vaccinated and unvaccinated volunteers. Green lines: significant differences between CD4+CD103+ and CD4+CD103− T_RM_ subset responses. A trend is represented by its p-value. Horizontal bars (black and red) represent median values.

**Additional file 5: Figure S5.** Terminal ileum LPMC *S*. Typhi-specific CD4+CD103+ and CD4+CD103− T_RM_ responses in Ty21a immunized and unimmunized healthy adults following stimulation with a Ty21a homogenate antigen preparation. Terminal ileum LPMC CD4+CD103+ and CD4+CD103− T_RM_ cells were stimulated with a Ty21a homogenate preparation (10 μg/mL). The net percentages of Ty21a homogenate (with media subtracted) *S*. Typhi-specific responses **(**IFNγ, IL-17A, IL-2, and TNFα) in (A) CD4+CD103+ and (B) CD4+CD103− T_RM_ subsets were compared between Ty21a vaccinated (n = 8; red symbols) and unvaccinated volunteers (n = 8; black symbols) with significant differences shown (*p < 0.05; **p < 0.005). Horizontal bars (black and red) represent median values.

**Additional file 6: Figure S6.** Absolute numbers of viable terminal ileum IEL and LPMC cell yields obtained from Ty21a vaccinated and unvaccinated volunteers. (A) Terminal ileum intraepithelial lymphocytes (IEL) and lamina propria mononuclear cells (LPMC) were isolated using an optimized method. The number of freshly isolated terminal ileum IEL and LPMC obtained from biopsies of Ty21a-vaccinated (n = 17; red symbols) and unvaccinated (n = 17; black symbols) volunteers were compared.

**Additional file 7: Figure S7.** Oral Ty21a immunization induces differential activation of terminal ileum IEL CD4+ T_RM_ subset producing single cytokines following anti-CD3/CD28 stimulation. Following anti-CD3/CD28 stimulation, CD4+CD103+ and CD4+CD103− T_RM_ subsets cytokine responses were stratified into multifunctional (MF) and single-positive effectors (S). Comparison of TI IEL CD4+CD103+ and CD4+CD103− T_RM_ subsets responses in (A) INFγ+; (B) IL-17A+; (C) IL-2+, and (D) TNFα+ MF and S in Ty21a-vaccinated (n = 10; red symbols) and unvaccinated volunteers (n = 8; black symbols) were determined with significant differences shown (*p < 0.05 ). Black lines: differences between Ty21a vaccinated and unvaccinated volunteers. Green lines: differences between CD4+CD103+ and CD4+CD103− T_RM_ subsets responses. Trends are represented by their p-values. Horizontal bars (black and red) represent median values. .

**Additional file 8: Figure S8.***S*. Typhi-specific responses of terminal ileum IEL CD4+ T_RM_ subsets in healthy adults following oral Ty21a immunization. The net percentages of *S*. Typhi-specific responses elicited by *S.* Typhi-infected autologous targets (IFNγ, IL-17A, IL-2, and TNFα) in terminal ileum IEL (A) CD4+CD103+ and (B) CD4+CD103− T_RM_ subsets were determined and compared between TI IEL obtained from Ty21a-vaccinated (n = 7; red symbols) and unvaccinated volunteers (n = 6; black symbols). A trend (p = 0.06) is indicated for IFNγ and TNFα in CD4+CD103− T_RM_ cells. Horizontal bars (black and red) represent median values.

**Additional file 9: Figure S9.** Expression of CD103 and CD69 in unstimulated LPMC and IEL CD4+ T_RM_ following oral Ty21a immunization. Cytograms show the expression levels of (A) CD103 and (C) CD69 in LPMC and IEL CD4+CD103+ and CD4+CD103− T_RM_ obtained from a representative volunteer. Mean fluorescence intensities (MFI) of CD103 and CD69 were determined in CD4+ T_RM_ subsets obtained from LPMC and IEL. Comparisons of (B) CD103 and (D) CD69 MFI expression on IEL and LPMC CD4+ T_RM_ subsets obtained from Ty21a-vaccinated (red; n = 8) and unvaccinated (black; n = 8) volunteers. Significant differences are shown (*p < 0.05; **p < 0.005). Trends are denoted with their p-value. Horizontal bars represent median values.

**Additional file 10: Table S1.** Spearman correlation analysis of LPMC and CD4+T_RM_ S. Typhi specific responses in unvaccinated and Ty21a vaccinated volunteers.

## Data Availability

The datasets supporting the findings of this study are available within the article and its Additional information files.

## Abbreviations

PBMC: Peripheral blood mononuclear cells
LPMC: Lamina propria mononuclear cells
IEL: Intraepithelial lymphocytes
TRM: Tissue resident memory T cell
EBV-B: Epstein–Barr virus (EBV)-transformed lymphoblastoid B cells
MF: *S.* Typhi-specific multifunctional cells
S: *S.* Typhi-specific single producing cells
TI: Terminal ileum
T-effector/memory: T_EM_ (CD62L^−^CD45RA^−^)
T-central/memory: T_CM_ (CD62L^+^CD45RA^−^)
T_EM_-CD45RA^+^: T_EMRA_(CD62L^−^CD45RA^+^)
LP: Lamina propria
