# Supplementary figures and images for: Oral typhoid vaccine Ty21a elicits antigen-specific resident memory CD4+ T cells in the human terminal ileum lamina propria and epithelial compartments
Source: J Transl Med. 2020 Feb 25;18:102. doi: 10.1186/s12967-020-02263-6 (PMC7043047; doi:10.1186/s12967-020-02263-6)

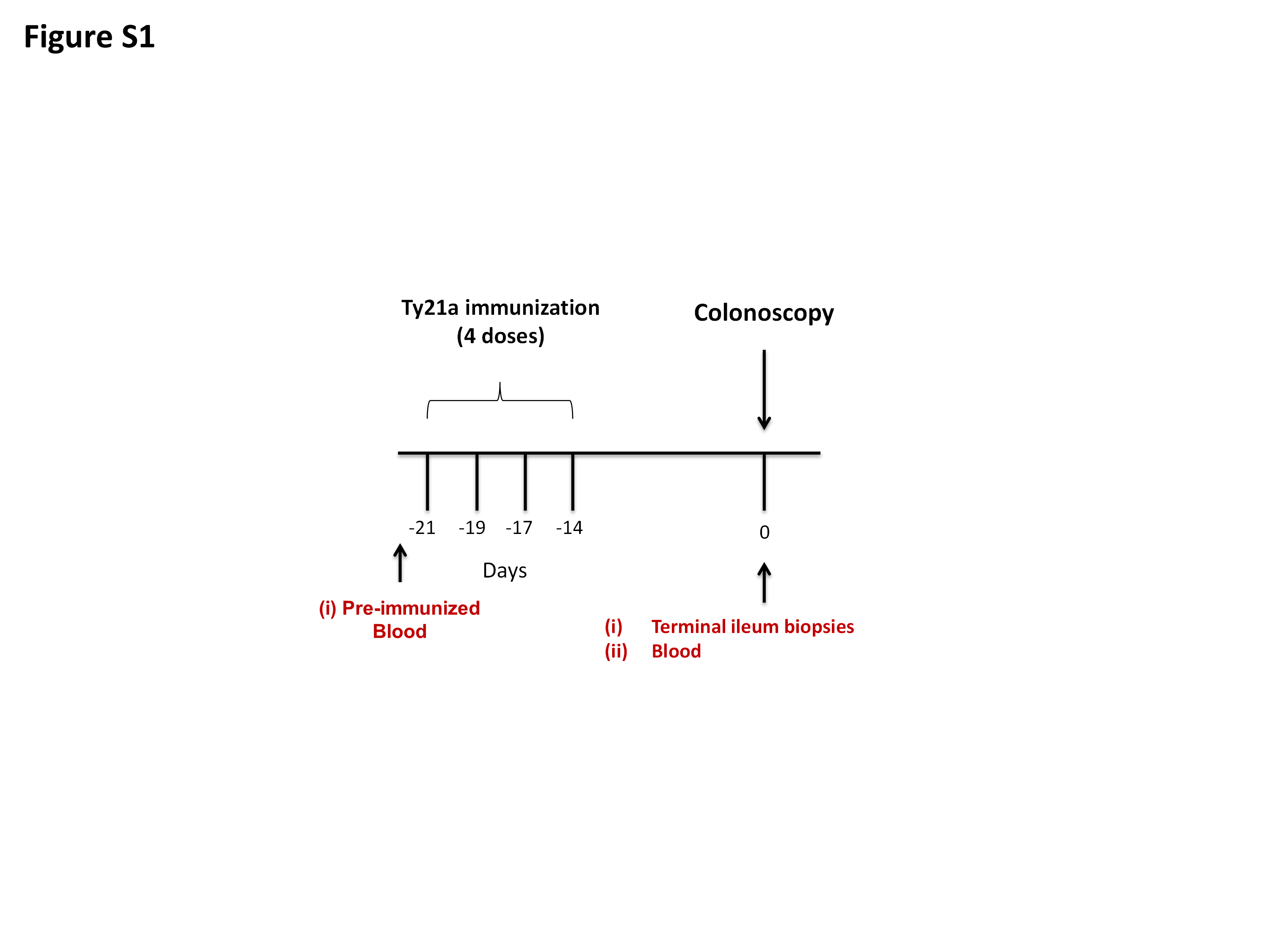

Supplement: Supplementary file 1 — Additional file 1: Figure S1. Study design. Oral typhoid vaccine Ty21a dose schedule (4 doses at -21 to -14 days) and time of collection of specimens (blood and terminal ileum (TI) biopsies) from volunteers undergoing routine screening colonoscopies. Autologous EBV-B cells were generated from pre-immunization blood. [file 12967_2020_2263_MOESM1_ESM.tif]

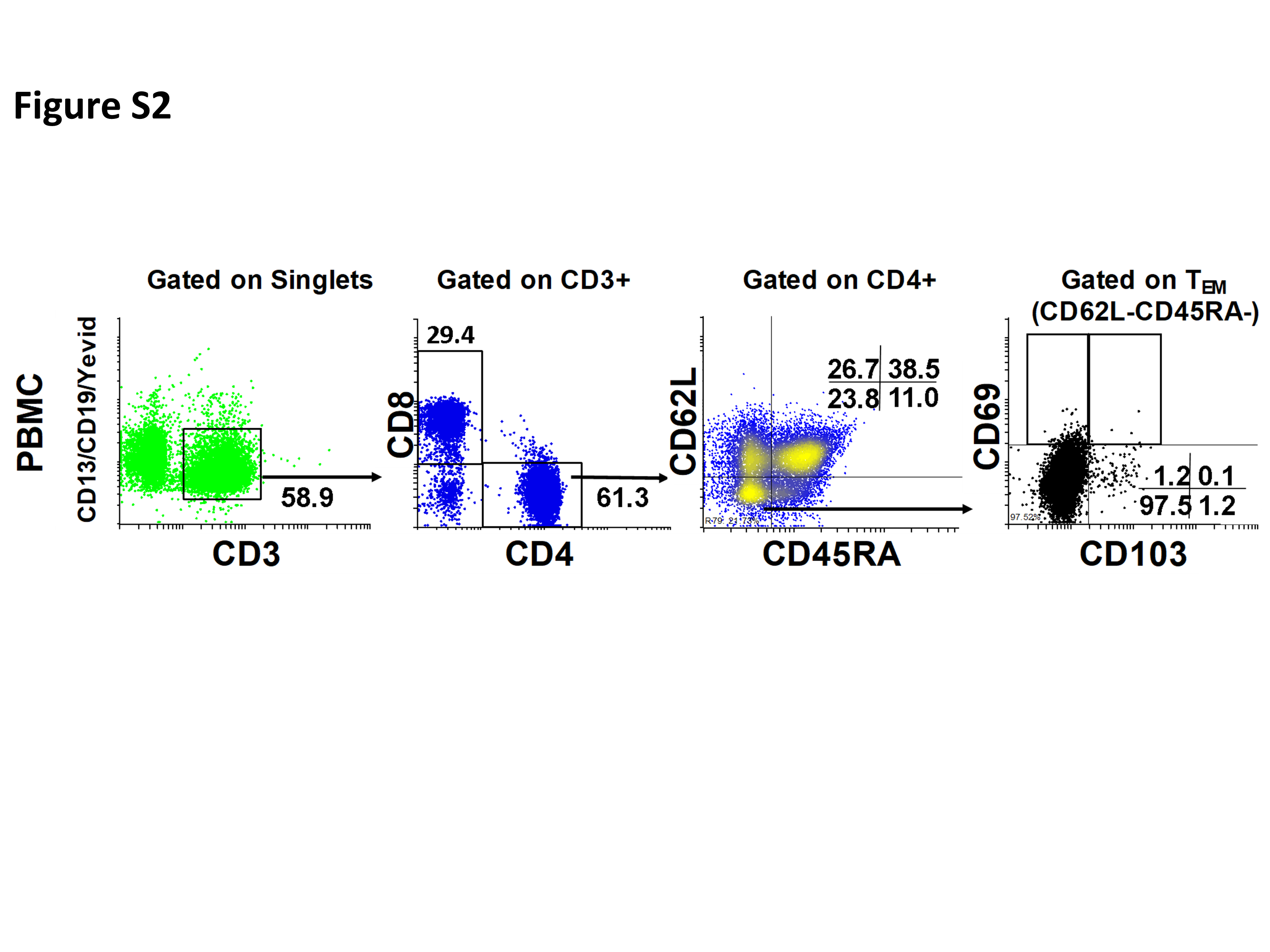

Supplement: Supplementary file 2 — Additional file 2: Figure S2. Gating Strategy for the measurement of CD4+ TRM cells in PBMC. PBMC were stained for T memory cell (TM) cell subsets, as well as CD69 and CD103 markers, and analyzed following the gating strategy shown. [file 12967_2020_2263_MOESM2_ESM.tif]

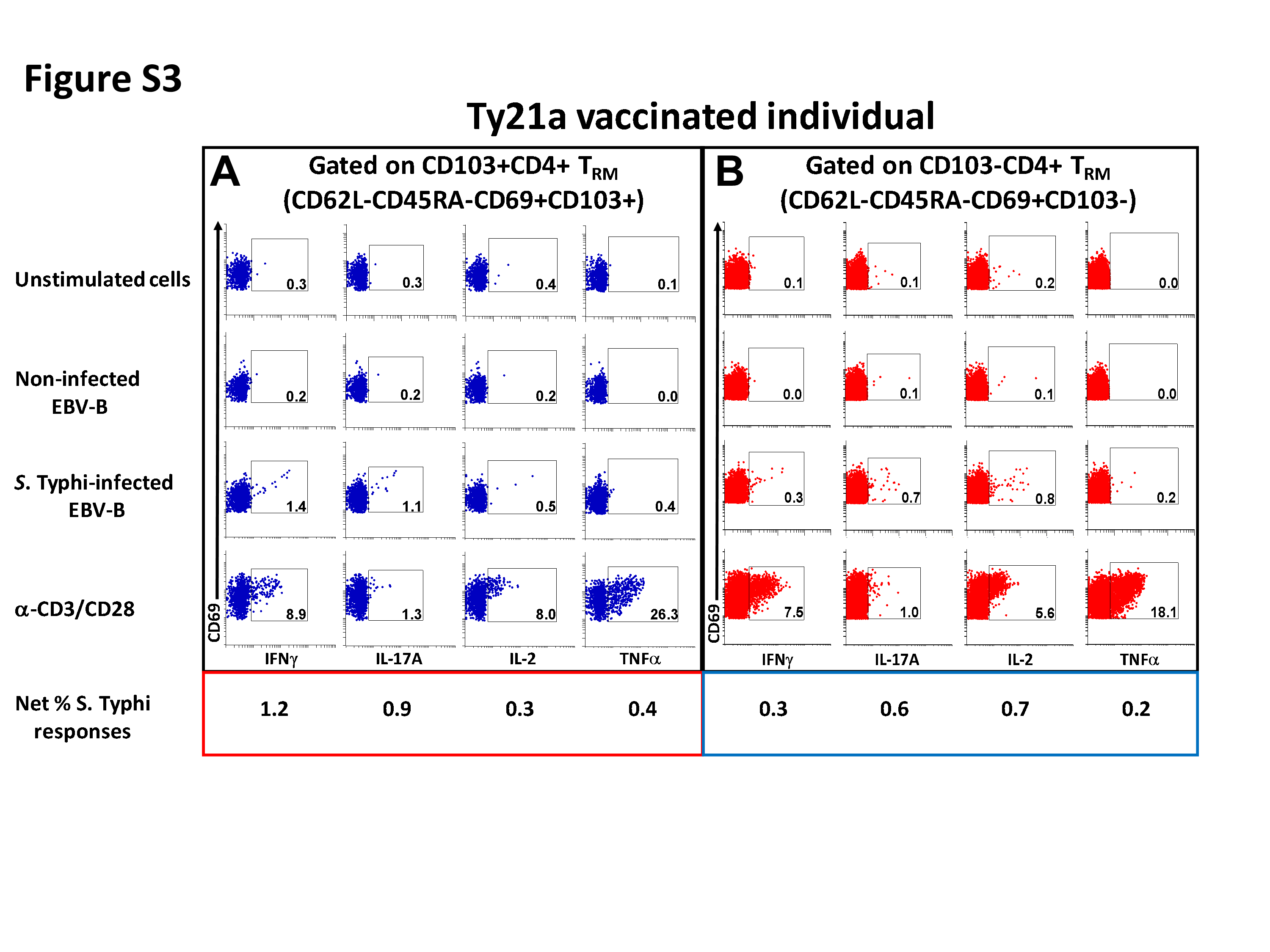

Supplement: Supplementary file 3 — Additional file 3: Figure S3. Activation of LPMC CD4+ TRM cell subsets isolated from terminal ileum of a Ty21a-vaccinated representative volunteer. (A) CD4+CD69+CD103+ TRM and (B) CD4+CD69+CD103− T cells were stimulated with non-infected or S. Typhi-infected autologous EBV-B cells and produced cytokines (IFNγ, IL-17A, IL-2 and TNFα) evaluated. Anti (α)-CD3/CD28 stimulation was used as a positive control in both subsets while unstimulated LPMC CD4+CD69+CD103+ or CD4+CD69+CD103− TRM alone (unstimulated) were used as negative controls. The percentage of positive cells in the gated regions is shown above the corresponding black boxes. Net % increases in S. Typhi responses (S. Typhi-infected EBV-B minus non-infected EBV-B) are shown in the boxes below. [file 12967_2020_2263_MOESM3_ESM.tif]

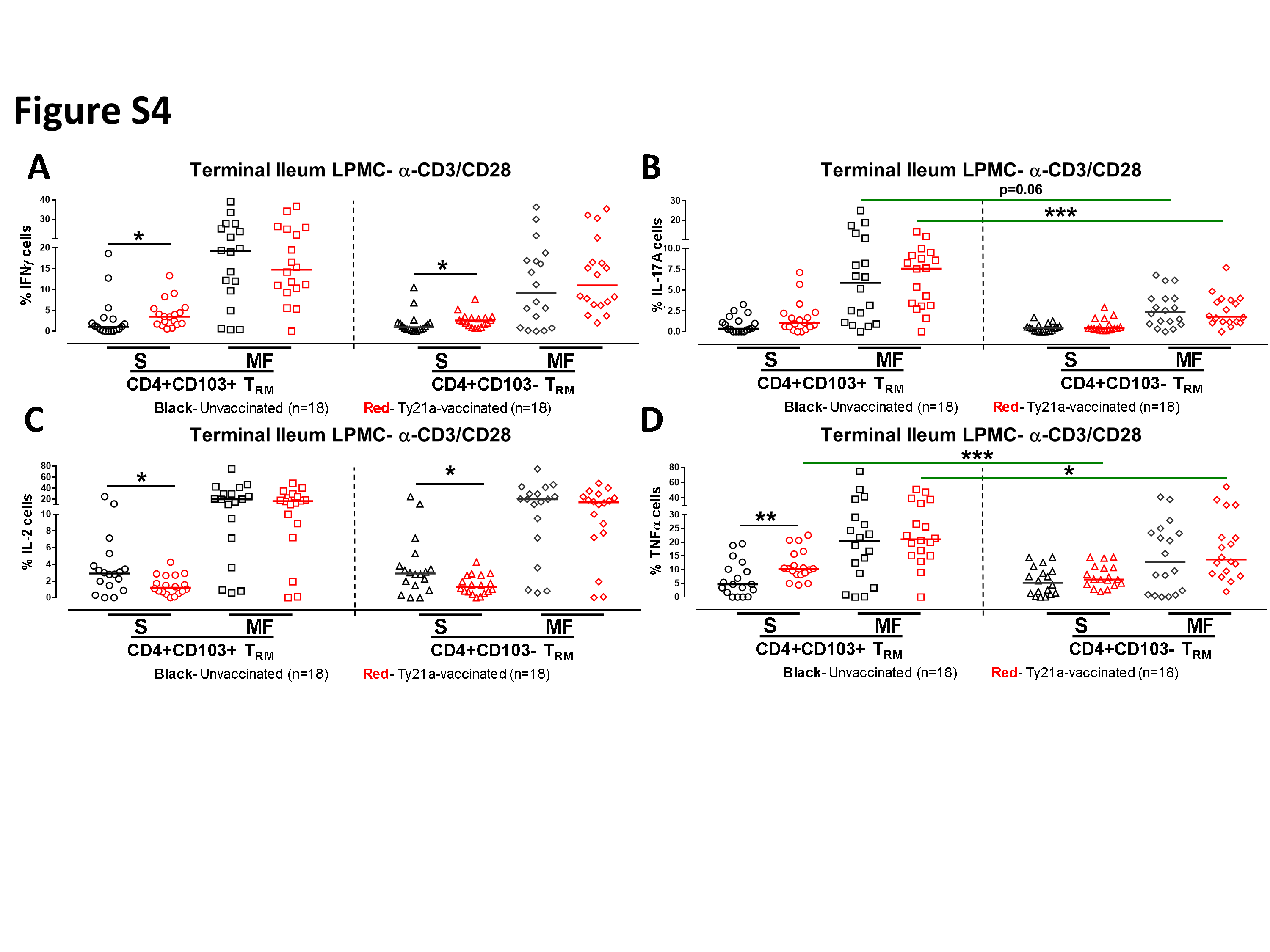

Supplement: Supplementary file 4 — Additional file 4: Figure S4. Oral Ty21a-immunization induces differential activation on terminal ileum LPMC CD4+ TRM subsets single cytokine responses following anti-CD3/CD28 stimulation. Following anti-CD3/CD28 stimulation, CD4+CD103+ and CD4+CD103− TRM subset cytokine responses were stratified into multifunctional (MF) and single-positive effectors (S). Comparison of TI LPMC CD4+CD103+ and CD4+CD103− TRM subsets responses in (A) INFγ+; (B) IL-17A+; (C) IL-2+, and (D) TNFα+ MF and S in Ty21a-vaccinated (n=18; red symbols) and unvaccinated volunteers (n=18; black symbols) were determined with significant differences shown (*p < 0.05; **p < 0.005; ***p < 0.0005). Black lines: significant differences between Ty21a vaccinated and unvaccinated volunteers. Green lines: significant differences between CD4+CD103+ and CD4+CD103− TRM subset responses. A trend is represented by its p-value. Horizontal bars (black and red) represent median values. [file 12967_2020_2263_MOESM4_ESM.tif]

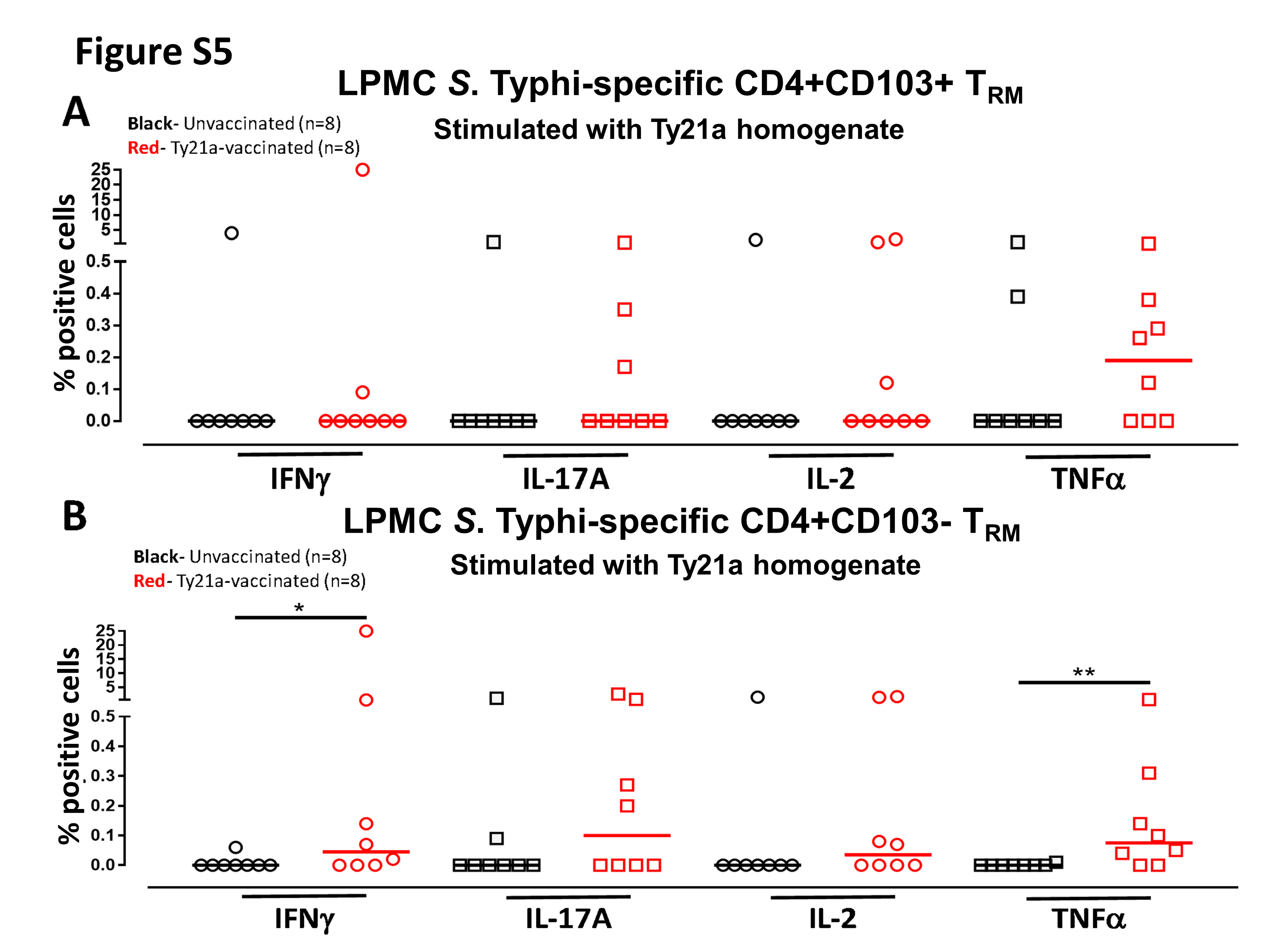

Supplement: Supplementary file 5 — Additional file 5: Figure S5. Terminal ileum LPMC S. Typhi-specific CD4+CD103+ and CD4+CD103− TRM responses in Ty21a immunized and unimmunized healthy adults following stimulation with a Ty21a homogenate antigen preparation. Terminal ileum LPMC CD4+CD103+ and CD4+CD103− TRM cells were stimulated with a Ty21a homogenate preparation (10 μg/mL). The net percentages of Ty21a homogenate (with media subtracted) S. Typhi-specific responses (IFNγ, IL-17A, IL-2, and TNFα) in (A) CD4+CD103+ and (B) CD4+CD103− TRM subsets were compared between Ty21a vaccinated (n = 8; red symbols) and unvaccinated volunteers (n = 8; black symbols) with significant differences shown (*p < 0.05; **p < 0.005). Horizontal bars (black and red) represent median values. [file 12967_2020_2263_MOESM5_ESM.tif]

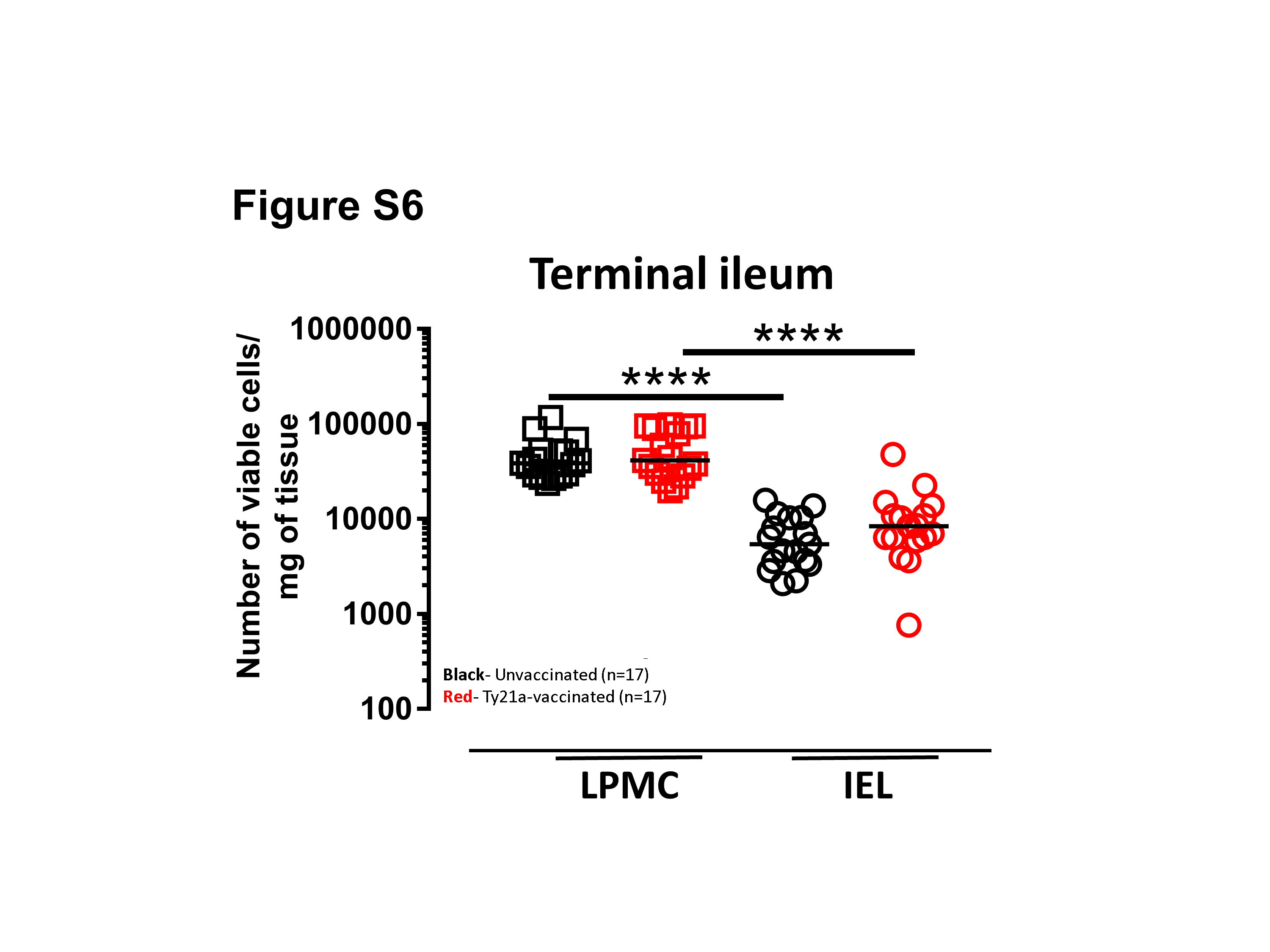

Supplement: Supplementary file 6 — Additional file 6: Figure S6. Absolute numbers of viable terminal ileum IEL and LPMC cell yields obtained from Ty21a vaccinated and unvaccinated volunteers. (A) Terminal ileum intraepithelial lymphocytes (IEL) and lamina propria mononuclear cells (LPMC) were isolated using an optimized method. The number of freshly isolated terminal ileum IEL and LPMC obtained from biopsies of Ty21a-vaccinated (n = 17; red symbols) and unvaccinated (n = 17; black symbols) volunteers were compared. [file 12967_2020_2263_MOESM6_ESM.tif]

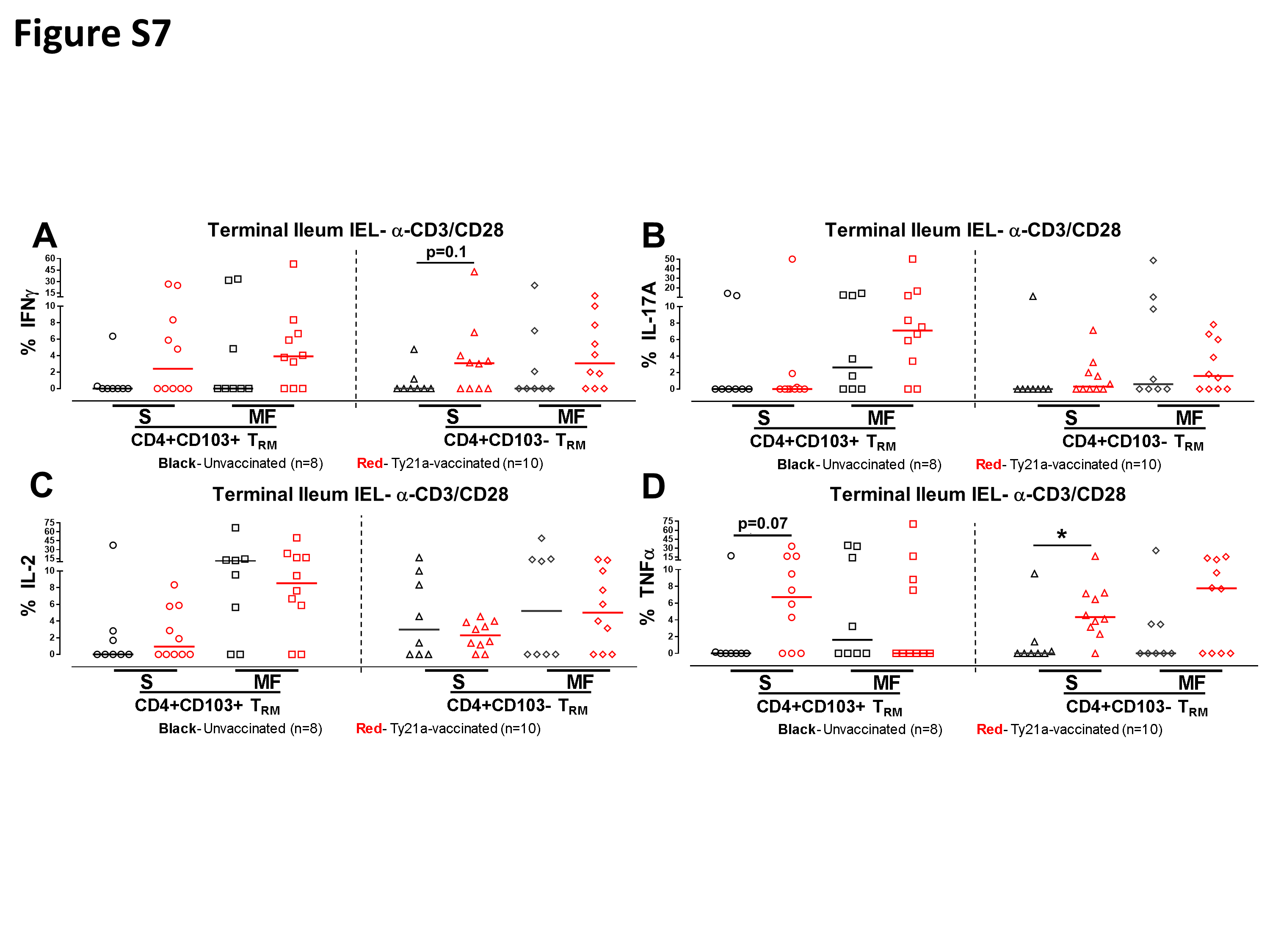

Supplement: Supplementary file 7 — Additional file 7: Figure S7. Oral Ty21a immunization induces differential activation of terminal ileum IEL CD4+ TRM subset producing single cytokines following anti-CD3/CD28 stimulation. Following anti-CD3/CD28 stimulation, CD4+CD103+ and CD4+CD103− TRM subsets cytokine responses were stratified into multifunctional (MF) and single-positive effectors (S). Comparison of TI IEL CD4+CD103+ and CD4+CD103− TRM subsets responses in (A) INFγ+; (B) IL-17A+; (C) IL-2+, and (D) TNFα+ MF and S in Ty21a-vaccinated (n = 10; red symbols) and unvaccinated volunteers (n = 8; black symbols) were determined with significant differences shown (*p < 0.05 ). Black lines: differences between Ty21a vaccinated and unvaccinated volunteers. Green lines: differences between CD4+CD103+ and CD4+CD103− TRM subsets responses. Trends are represented by their p-values. Horizontal bars (black and red) represent median values. . [file 12967_2020_2263_MOESM7_ESM.tif]

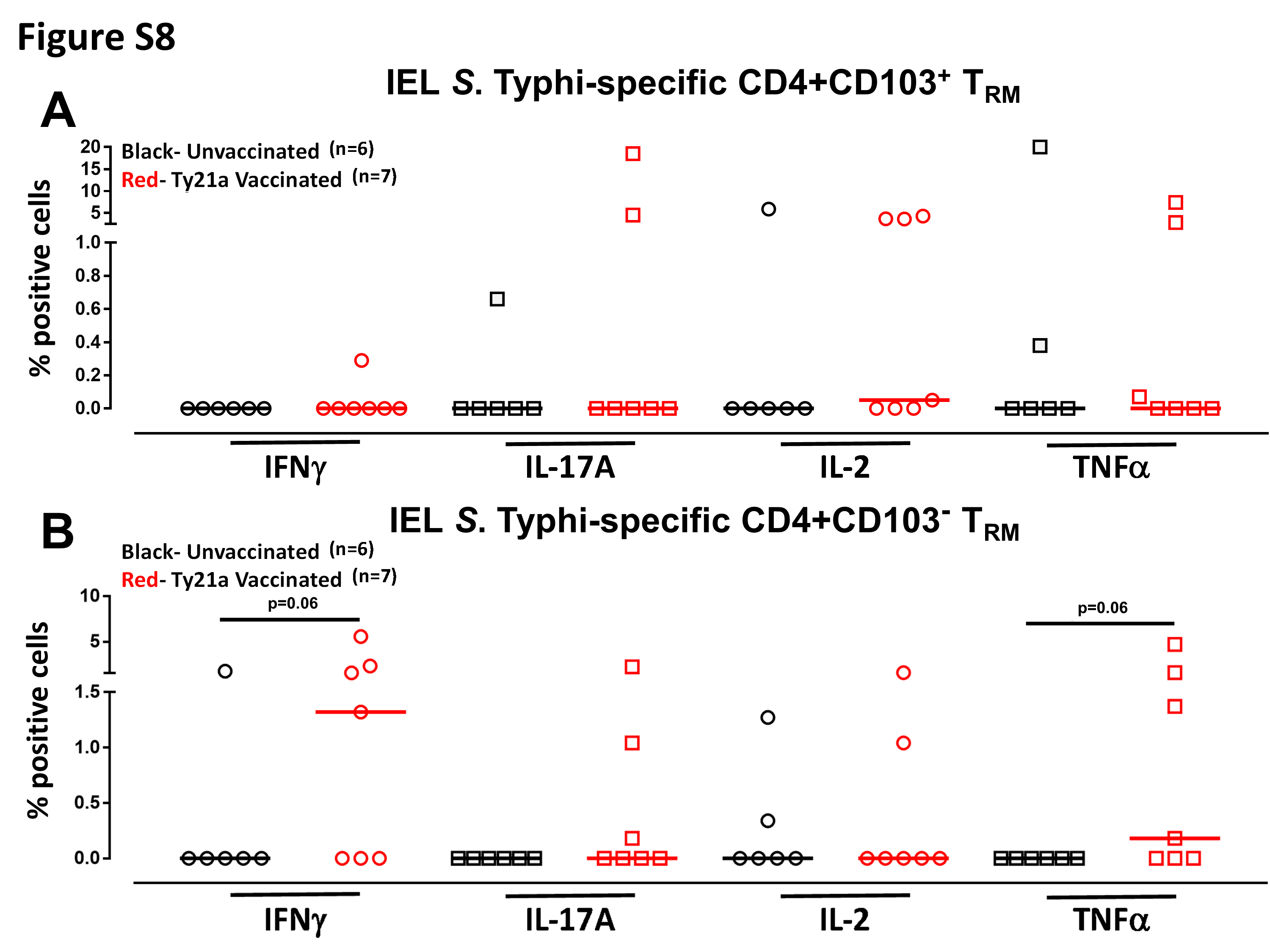

Supplement: Supplementary file 8 — Additional file 8: Figure S8.S. Typhi-specific responses of terminal ileum IEL CD4+ TRM subsets in healthy adults following oral Ty21a immunization. The net percentages of S. Typhi-specific responses elicited by S. Typhi-infected autologous targets (IFNγ, IL-17A, IL-2, and TNFα) in terminal ileum IEL (A) CD4+CD103+ and (B) CD4+CD103− TRM subsets were determined and compared between TI IEL obtained from Ty21a-vaccinated (n = 7; red symbols) and unvaccinated volunteers (n = 6; black symbols). A trend (p = 0.06) is indicated for IFNγ and TNFα in CD4+CD103− TRM cells. Horizontal bars (black and red) represent median values. [file 12967_2020_2263_MOESM8_ESM.tif]

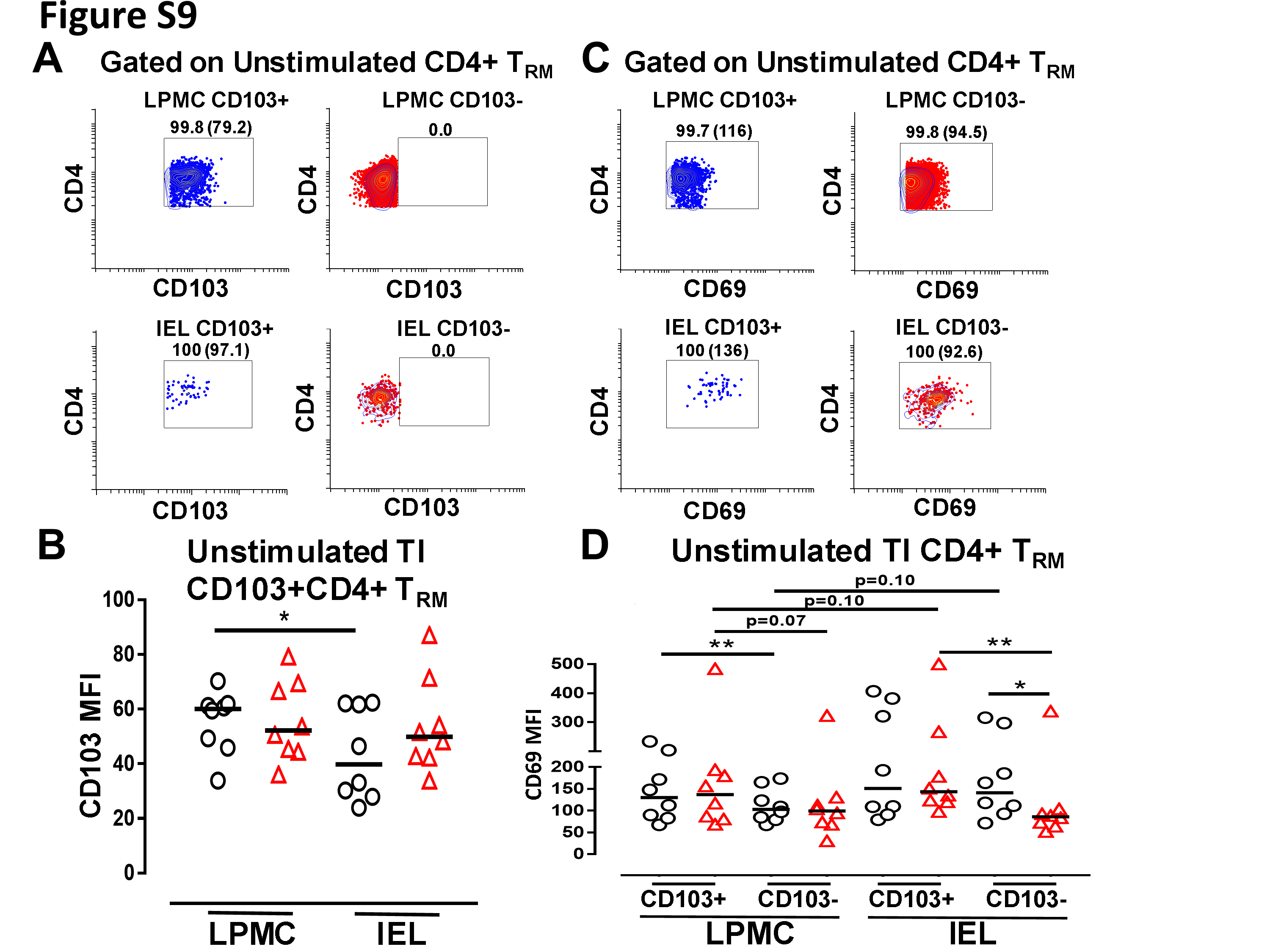

Supplement: Supplementary file 9 — Additional file 9: Figure S9. Expression of CD103 and CD69 in unstimulated LPMC and IEL CD4+ TRM following oral Ty21a immunization. Cytograms show the expression levels of (A) CD103 and (C) CD69 in LPMC and IEL CD4+CD103+ and CD4+CD103− TRM obtained from a representative volunteer. Mean fluorescence intensities (MFI) of CD103 and CD69 were determined in CD4+ TRM subsets obtained from LPMC and IEL. Comparisons of (B) CD103 and (D) CD69 MFI expression on IEL and LPMC CD4+ TRM subsets obtained from Ty21a-vaccinated (red; n = 8) and unvaccinated (black; n = 8) volunteers. Significant differences are shown (*p < 0.05; **p < 0.005). Trends are denoted with their p-value. Horizontal bars represent median values. [file 12967_2020_2263_MOESM9_ESM.tif]

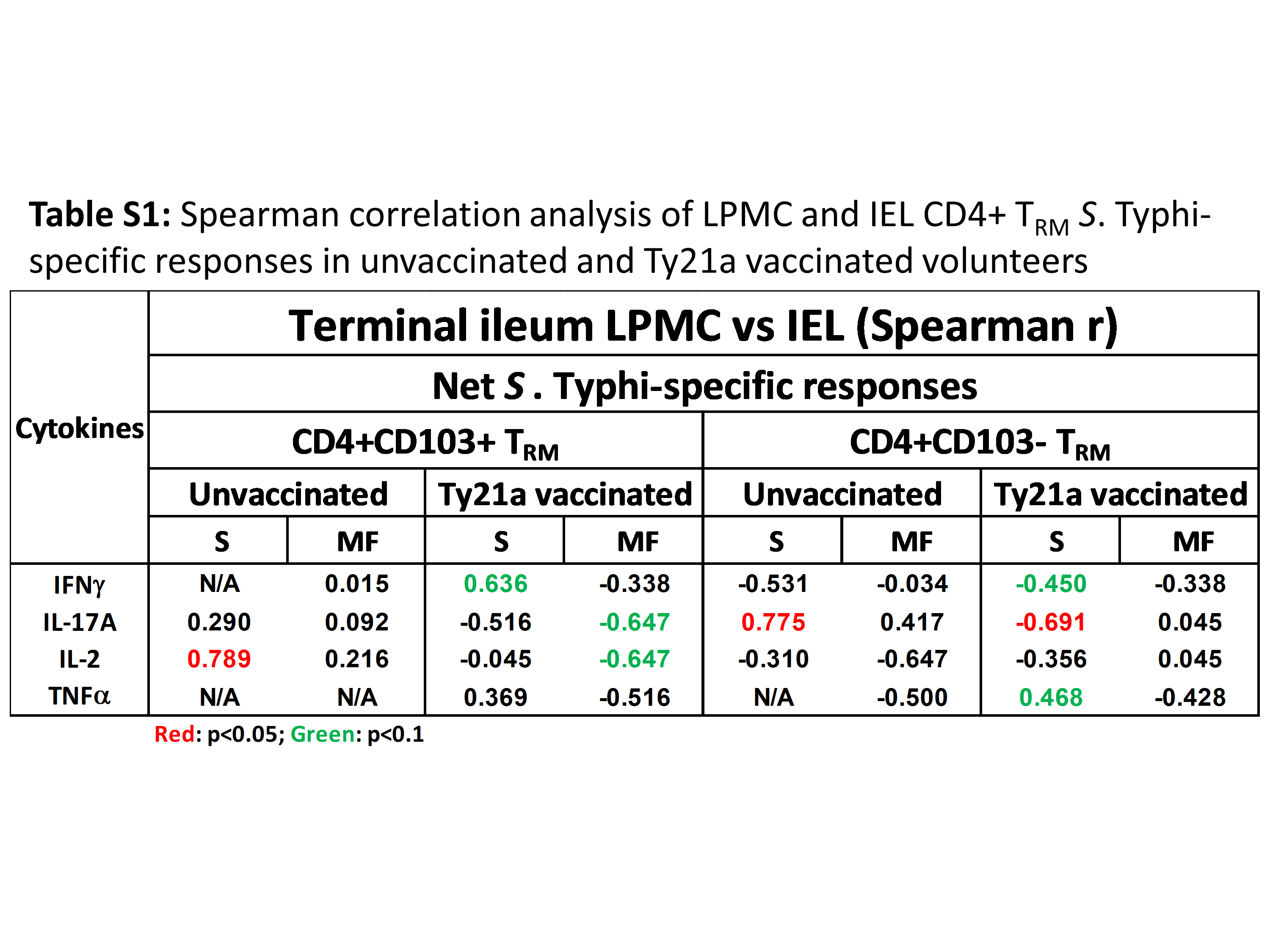

Supplement: Supplementary file 10 — Additional file 10: Table S1. Spearman correlation analysis of LPMC and CD4+TRM S. Typhi specific responses in unvaccinated and Ty21a vaccinated volunteers. [file 12967_2020_2263_MOESM10_ESM.tif]
